# Plasma level of omentin-1, its expression, and its regulation by gonadotropin-releasing hormone and gonadotropins in porcine anterior pituitary cells

**DOI:** 10.1038/s41598-023-46742-4

**Published:** 2023-11-07

**Authors:** Natalia Respekta, Karolina Pich, Ewa Mlyczyńska, Kamil Dobrzyń, Christelle Ramé, Tadeusz Kamiński, Nina Smolińska, Joëlle Dupont, Agnieszka Rak

**Affiliations:** 1 Laboratory of Physiology and Toxicology of Reproduction, Institute of Zoology and Biomedical Research, Jagiellonian University, Gronostajowa 9 Street, 30-387 Kraków, Poland; 2 Doctoral School of Exact and Natural Sciences, Jagiellonian University, Kraków, Poland; 3 Department of Zoology, Faculty of Biology and Biotechnology, University of Warmia and Mazury, Kortowo, Olsztyn, Poland; 4 INRAE, UMR85, Unité Physiologie de la Reproduction et des Comportements, Nouzilly, France; 5 Department of Animal Anatomy and Physiology, Faculty of Biology and Biotechnology, University of Warmia and Mazury, Kortowo, Olsztyn, Poland

**Keywords:** Cell biology, Physiology, Endocrinology

## Abstract

Omentin-1 (OMNT1) is an adipokine involved in the regulation of energy metabolism, insulin sensitivity, and reproduction. The present study was the first to investigate the plasma levels and expression of OMNT1 in the anterior pituitary (AP) gland on days 2–3, 10–12, 14–16, and 17–19 of the estrous cycle of normal-weight Large White (LW) and fat Meishan (MS) pigs. Next, we determined the effect of GnRH, LH, and FSH on the OMNT1 levels in cultured AP cells. The gene and protein expression of OMNT1 in AP fluctuated during the estrous cycle, with a higher expression in MS than in LW (except on days 10–12). However, plasma levels of OMNT1 were higher in LW than in MS. OMNT1 was localized in somatotrophs, lactotrophs, thyrotrophs, and gonadotrophs. In LW pituitary cells, GnRH and gonadotropins stimulated OMNT1 protein expression (except FSH on days 14–16) and had no effect on OMNT1 levels in the culture medium. In MS pituitary cells, we observed that GnRH and LH increased while FSH decreased OMNT1 protein expression. These findings showed OMNT1 expression and regulation in the porcine AP and suggested that OMNT1 could be a new player modifying the pituitary functions.

## Introduction

Omentin-1 (OMNT1), also known as intelectin-1 (ITLN1), is an adipokine that is regarded as a secretory protein produced by adipose tissue^1^. It was first described in mouse Paneth cells as a lectin involved in intestinal defense mechanisms against pathogenic bacteria^2,3^ and also as an adipokine originally identified from omental adipose tissue^4^. OMNT1 is encoded by the *ITLN1* gene, which, in humans, lies in the chromosomal region 1q21.3. The protein sequence comprises 313 amino acids, including a highly hydrophobic signal peptide of 18 amino acids, and has a total molecular weight of 34 kDa^4^. Accordingly, OMNT1 is the major circulating form of OMNT^5^. It has 83% homology with OMNT2, which is secreted primarily into the intestinal lumen as an innate defense against parasites in intestinal diseases^6^. In pigs, this protein is described as OMNT2 in the extracellular region, and its sequence has 324 amino acids (UniProt) and 82% homology with human OMNT1^7^. Until now, the specific receptor for OMNT1 has not been identified. However, the latest data suggest that OMNT1 has a fibrinogen-like domain^8^ and acts by binding to the integrin receptors αvβ3 and αvβ5 in mouse macrophages^9^. On the other hand, OMNT1 modulates the phosphorylation of the insulin receptor and the beta subunit of the insulin-like growth factor-1 (IGF-1) receptor in human granulosa cells^10^. Several lines of experimental and clinical evidence indicate that OMNT1 may mediate cardiovascular protective effects and that circulating OMNT1 levels can be used as a biomarker of cancers, polycystic ovary syndrome, preeclampsia, inflammatory diseases, and metabolic disorders, including diabetes and metabolic syndrome^1^. Moreover, OMNT1 can promote inflammation in primary human adipocytes through the activation of extracellular signal-regulated kinase^11^, and it increases insulin-stimulated glucose uptake in human adipocytes^12^ via the activation of phosphatidylinositol 3-kinase/protein kinase B^13^. Literature data confirmed the orexigenic effect of OMNT1 administered intraperitoneally to rats, probably due to decreased gene expression of cocaine- and amphetamine-regulated transcript (CART) and corticotropin-releasing hormone (CRH) and increased synthesis and release of norepinephrine in the hypothalamus^14^. Also, Cloix et al.^10^ described OMNT1's role in human granulosa cells; they observed that OMNT1 increased IGF-1-induced progesterone (P_4_) and estradiol (E_2_) secretion, and this was associated with an increase in the levels of steroidogenic acute regulatory protein and cytochrome P450 family 19 subfamily A member 1, as well as an increase in IGF-1R signaling.

The highest expression of OMNT1 was described not only in visceral adipose stromal-vascular cells^12^ but also in human epicardial adipose tissue, peri-internal mammary gland tissue, the human placenta and ovary^10,15–17^, porcine peri-renal adipose tissue^18^, and the sheep ovary^19^. Some studies showed that OMNT1 is present in cerebral microvasculature, neurons, and glial cells in the human mesencephalon^20^ and in the choroid plexus of newborn calves^21^. The level of plasma circulating OMNT1 is higher in women than in men^22^ and is negatively correlated with free testosterone, androgen, the ratio of luteinizing hormone/follicle-stimulating hormone (LH/FSH), tumor necrosis factor α, interleukin-6, and leptin^23,24^. Moreover, it is positively associated with adiponectin levels in normal and overweight patients^25^. Nevertheless, in human adipose tissue, OMNT1 is downregulated by glucose and insulin^26^ and upregulated by fibroblast growth factor-21 and dexamethasone^27,28^, suggesting hormonal regulation of OMNT1 expression. However, the effect of hormonal changes associated with the phases of the estrous cycle on OMNT1 levels has not been studied in pituitary cells. Interestingly, OMNT1 levels are strongly dependent on the body's energy status; lower plasma levels were observed in overweight and obese women than in women with a normal body weight^5^. Likewise, our previous data documented that plasma and peri-renal adipose tissue levels of OMNT1 were lower in fat Meishan (MS) pigs than in normal-weight Large White (LW) pigs^18^; however, the expression of OMNT1 in pituitary cells remains largely unexplored.

To date, we hypothesized that the plasma and pituitary levels of OMNT1 depend on the phase of the estrous cycle and the metabolic status in pigs and that these levels are regulated by gonadotropin-releasing hormone (GnRH) and the gonadotropins LH and FSH. Therefore, the aim of this study was to determine the plasma levels and the gene and protein expression of OMNT1 in the anterior pituitary (AP) gland of LW and MS pigs during the estrous cycle, as well as the cellular immunolocalization of OMNT1. Next, we determined the direct in vitro effect of GnRH and gonadotropins on OMNT1 levels in porcine AP cells. For animal models, we used two prolific breeds of pigs: fat MS pigs and normal-weight LW pigs, which differ in fat content (MS > LW). Obese MS pigs have fat cells that are similar in size and number to the fat cells in humans^29^. Furthermore, obese MS pigs have more adipose tissue and organ fat and reach sexual maturity at an earlier age than LW pigs^18,30,31^.

## Results

### Expression of OMNT1 in the porcine AP gland during the estrous cycle

**Figure 1:**
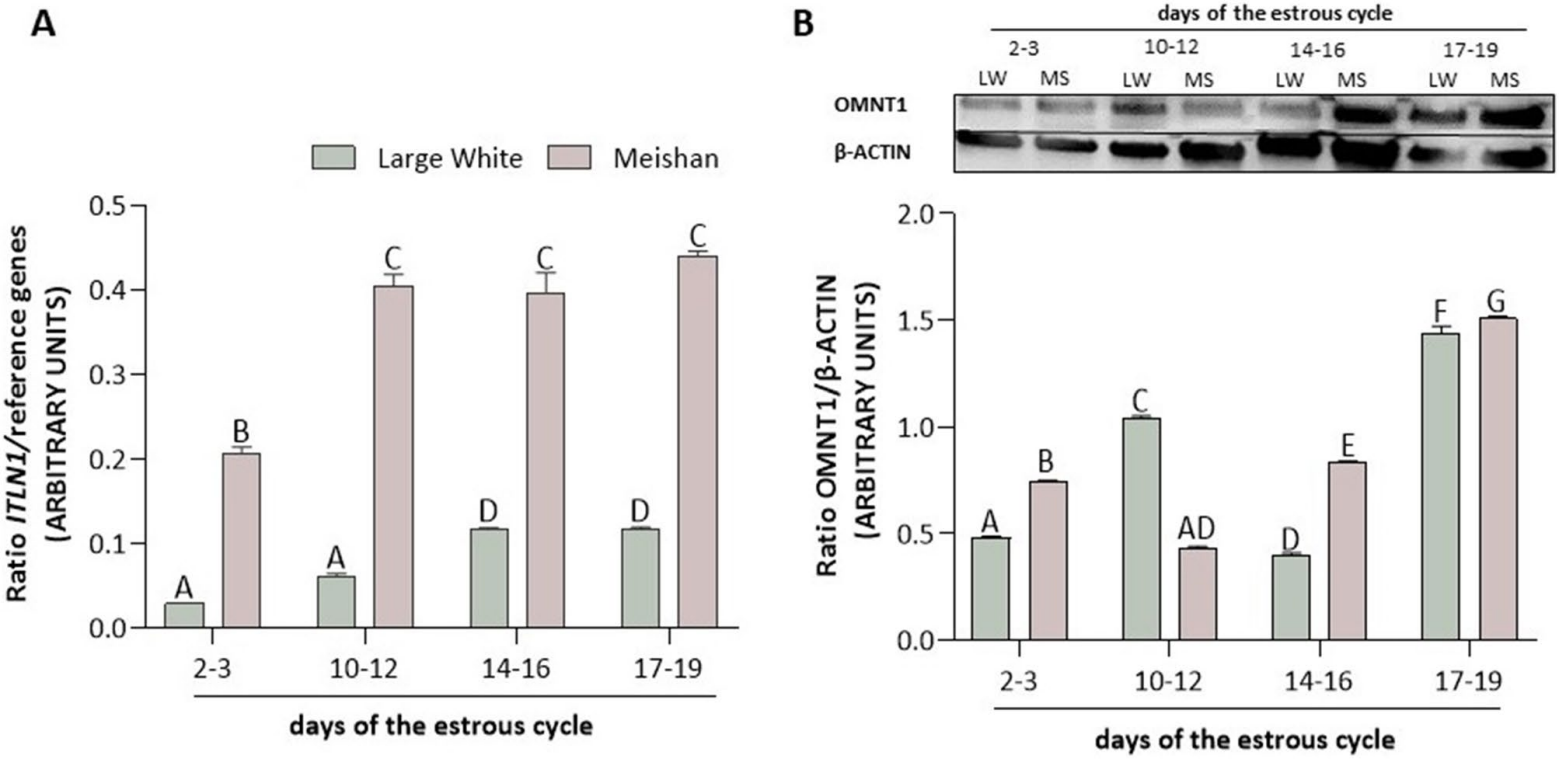
The gene (**A**) and protein (**B**) expression of omentin-1 (OMNT1) in the AP gland during the estrous cycle (days 2–3, 10–12, 14–16, 17–19) of Large White (LW) and Meishan (MS) pigs. The gene expression was analyzed using RT-qPCR, and the results were normalized by the geometric mean of the reference gene expression. The protein expression was analyzed using western blot, and the results are presented as a densitometric normalized ratio relative to the β-actin abundance. The results of at least six independent replicates are presented as means ± SEM for each group. Bars with different superscripts are significantly different (*p* < 0.05). Representative blots are attached as Supplementary Fig. 1.

In the pituitary gland of both LW and MS pigs, we noted that the expression of the *ITLN1* gene increased with the progression of the estrous cycle (*p* < 0.05; Fig. 1A). In LW pigs, a significant *ITLN1* increase was evident from days 14–16 of the estrous cycle, while in MS pigs the increase occurred from days 10–12 of the estrous cycle. We noted higher *ITLN1* gene expression in the pituitary gland of MS pigs than in that of LW pigs (*p* < 0.05; Fig. 1A). As shown in Fig. 1B, we found that in LW pigs, OMNT1 protein expression increased on days 10–12 of the estrous cycle, decreased on days 14–16 of the estrous cycle, and increased on days 17–19 of the estrous cycle (*p* < 0.05). In MS pigs, we observed that OMNT1 protein expression decreased on days 10–12 of the estrous cycle and then increased from days 14–16 to days 17–19 of the estrous cycle (*p* < 0.05). We noted that the protein expression of OMNT1 was higher in the pituitary gland of MS pigs than in that of LW pigs, except on days 10–12 of the estrous cycle (*p* < 0.05; Fig. 1B).

### Plasma concentrations of OMNT1, LH, FSH, P_4_, and E_2_ and their correlations

**Figure 2:**
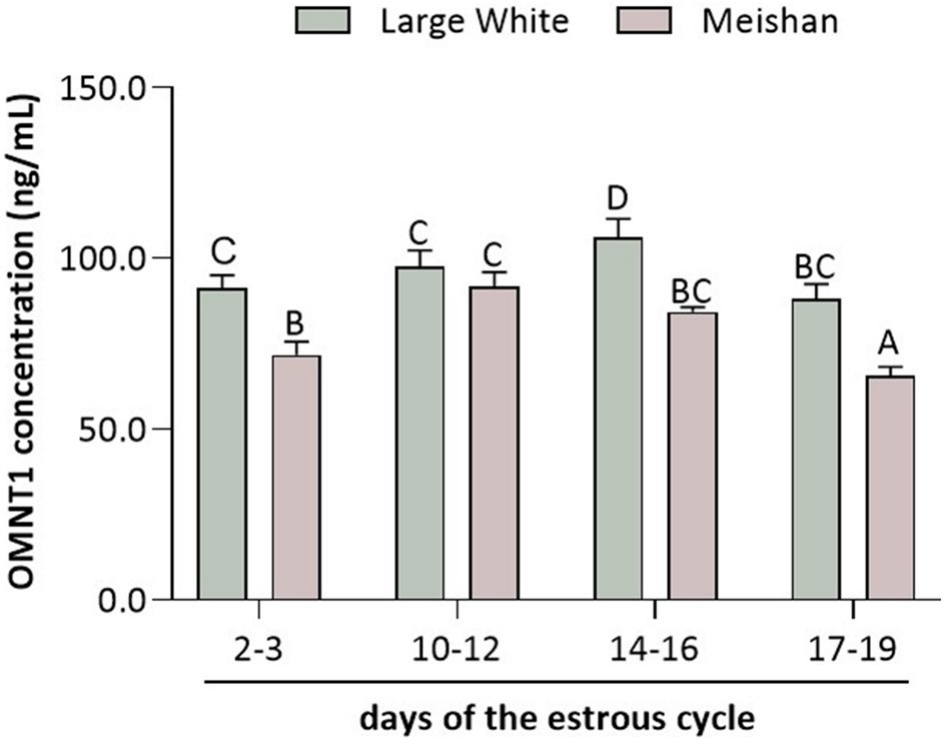
Plasma omentin-1 (OMNT1) concentration during the estrous cycle (days 2–3, 10–12, 14–16, 17–19) of Large White (LW) and Meishan (MS) pigs. The hormone concentration in blood plasma was evaluated using ELISA. The results of at least six independent replicates are presented as means ± SEM for each group. Bars with different superscripts are significantly different (*p* < 0.05).

During the estrous cycle, the highest plasma concentration of OMNT1 was observed on days 14–16 of the estrous cycle in LW pigs and on days 10–12 and 14–16 of the estrous cycle in MS pigs (*p* < 0.05; Fig. 2). As shown in Fig. 2, significantly higher plasma concentrations of OMNT1 were noted in LW pigs than in MS pigs (*p* < 0.05).

We also measured the concentration of gonadotropin and steroid hormones in plasma during the estrous cycle in LW and MS pigs (Supplementary Fig. 2). We demonstrated a positive correlation of *ITLN1* expression in the AP gland with the plasma level of LH in LW pigs (r = 0.610, *p* = 0.004). However, we observed a positive correlation between OMNT1 protein and LH levels in both pig breeds (LW: r = 0.597, *p* = 0.006, MS: r = 0.710, *p* = 0.001). Significant correlation between OMNT1 protein and FSH levels in MS (r = 0.462, *p* = 0.008) (Table 1) was also noted. Additionally, we showed the negative correlations between *ITLN1* expression with E_2_ concentration in LW (r = − 0.551, *p* = 0.027) (Table 1).

**Table 1:** Pearson correlation coefficient (r) calculated between LH (ng/mL), FSH (ng/mL), P_4_ (ng/mL) or E_2_ (pg/mL) and omentin-1 (OMNT1) (ng/mL) plasma concentration, OMNT1 gene and protein expression in AP gland of Large White (LW) (n = 6) and Meishan (MS) (n = 6) pigs.

	LW	MS
LH		
OMNT1 in plasma	r = − 0.318 *p* = 0.247	r = − 0.096 *p* = 0.705
*ITLN1* in AP	**r = 0.610** ***p* = 0.004**	r = 0.233 *p* = 0.296
OMNT1 in AP	**r = 0.597** ***p* = 0.006**	**r = 0.710** ***p* = 0.001**
FSH		
OMNT1 in plasma	r = 0.153 *p* = 0.573	r = − 0.391 *p* = 0.098
*ITLN1* in AP	r = 0.289 *p* = 0.216	r = − 0.150 *p* = 0.551
OMNT1 in AP	r = 0.179 *p* = 0.476	**r = 0.462** ***p* = 0.008**
OMNT1 in plasma	r = − 0.392 *p* = 0.233	r = − 0.171 *p* = 0.526
P_4_		
*ITLN1* in AP	r = − 0.311 *p* = 0.224	r = − 0.445 *p* = 0.064
OMNT1 in AP	r = -0.261 *p* = 0.296	r = − 0.421 *p* = 0.104
OMNT1 in plasma	r = − 0.212 *p* = 0.508	r = − 0.135 *p* = 0.561
E_2_		
*ITLN1* in AP	**r =** − **0.551** ***p* = 0.027**	r = 0.194 *p* = 0.471
OMNT1 in AP	r = − 0.437 *p* = 0.118	r = 0.354 *p* = 0.106

The correlation was noted as “r” and the *p* value (*p*) was considered significant if *p* < 0.05 (bold).

*LH* luteinizing hormone, *FSH* follicle stimulating hormone, *P*_*4*_ progesterone, *E*_*2*_ estradiol, *OMNT1* omentin-1, *ITLN1* intelectin-1, *AP* anterior pituitary, *LW* Large White, *MS* Meishan.

### Co-localization of OMNT1 with tropic hormones in the AP gland

**Figure 3:**
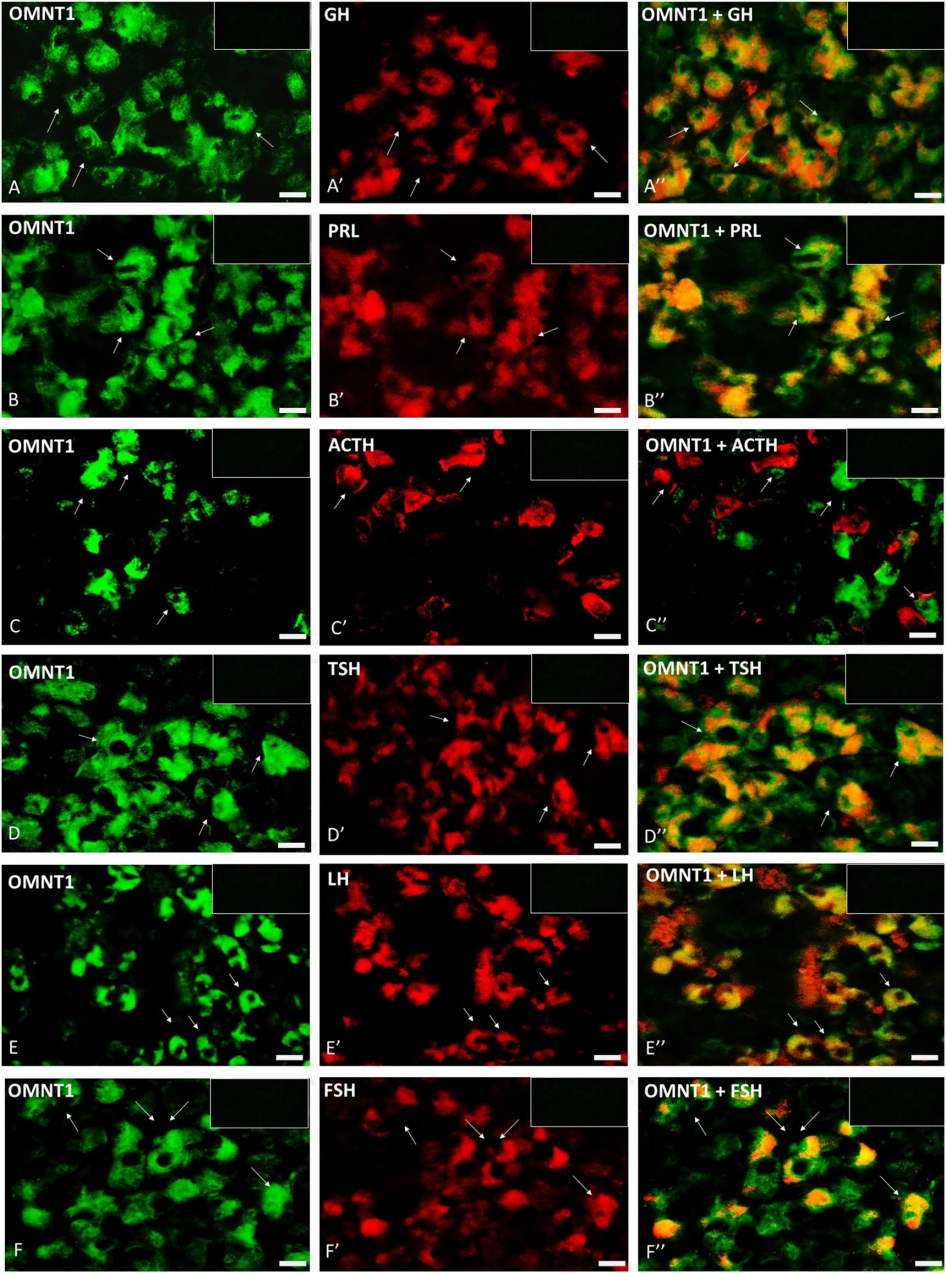
Co-localization of omentin-1 (OMNT1) in porcine tropic cells on days 10–12 of the estrous cycle in Large White pigs. The immunoreactivity of all hormones was determined by fluorescent immunohistochemistry. Left column: OMNT1 expression, visualized by Alexa Fluor 488 as green fluorescence; middle column: tropic hormone expression, visualized by Alexa Fluor 594 as red fluorescence; right column: immunofluorescent double labeling of OMNT1 and GH (**A**–**A**″), PRL (B-B”), ACTH (**C**–**C**″), TSH (**D**–**D**″), LH (**E**-**E**″), and FSH (**F**–**F**″). Arrows indicate examples of dual-labeled cells or lack of co-expression between OMNT1 and ACTH (**C**–**C′**). Scale bar: 20 μm. Section thickness 5 μm.

**Figure 4:**
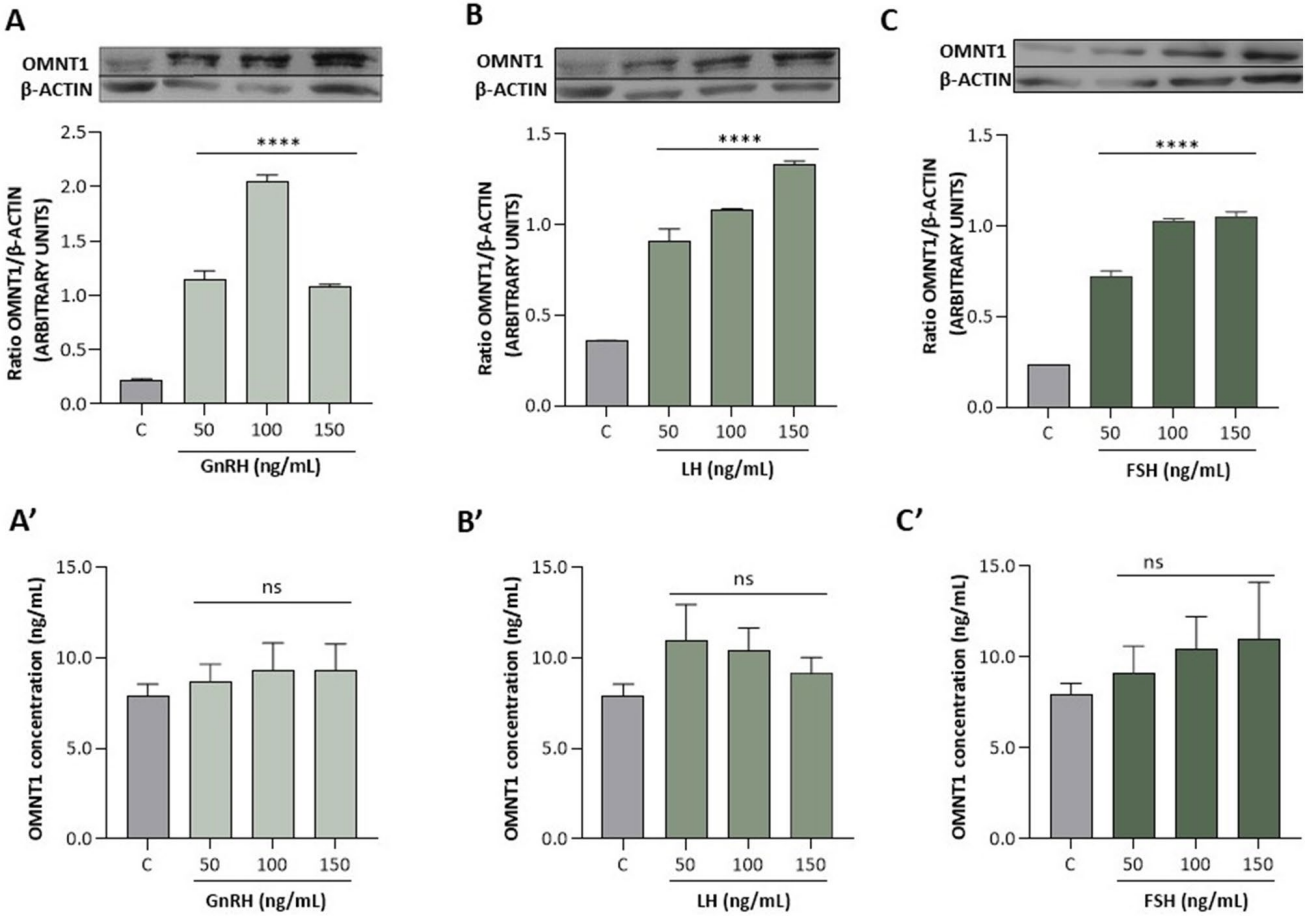
Effect of GnRH, LH, and FSH at doses of 50,100, and 150 ng/mL on omentin-1 (OMNT1) protein expression (**A**–**C**) and levels in the culture medium (**A**′–**C**′) of AP cells collected on days 10–12 of the estrous cycle of LW pigs. The protein expression was analyzed using western blot, and the results are presented as a densitometric normalized ratio relative to the β-actin abundance. The OMNT1 concentration in the culture medium was evaluated using ELISA. The results of at least four independent replicates are presented as means ± SEM for each group. Data were compared by an unpaired two-tailed Student's t test (****p < 0.0001). Representative blots are attached as Supplementary Fig. 1.

The immunofluorescence staining confirmed OMNT1 in porcine tropic cells on days 10–12 of the estrous cycle of LW pigs (Fig. 3). We observed an OMNT1 signal in the cytoplasm of immunoreactive (IR) cells: somatotrophs (Fig. 3A–A″), lactotrophs (Fig. 3B–B″), thyrotrophs (Fig. 3D–D″), and gonadotrophs (Figs. 3E–E″ and 4F–F″), responsible for the secretion of growth hormone (GH), prolactin (PRL), thyroid-stimulating hormone (TSH), and LH and FSH, respectively. Interestingly, the OMNT1 signal was not observed in corticotrophs, which are adrenocorticotropic hormone (ACTH)-IR cells (Fig. 3C–C″). In all negative controls, staining was absent when the primary antibody was replaced by a non-immune serum.

### In vitro effect of GnRH, LH, and FSH on OMNT1 levels in AP cells

We observed that for 24 h of cell culture, all doses of GnRH and gonadotropins (50–150 ng/mL) significantly increased OMNT1 protein expression in pituitary cells on days 10–12 of the estrous cycle in LW pigs (*p* < 0.05; Fig. 4A–C).

**Figure 5:**
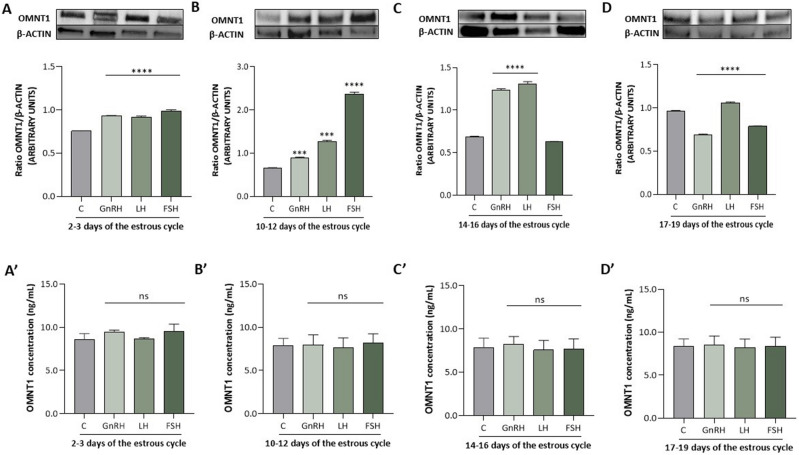
Effect of GnRH, LH, and FSH at 100 ng/mL on omentin-1 (OMNT1) protein expression (**A**–**C**) and levels in the culture medium (**A**′–**C**′) of AP cells collected on days 2–3, 10–12, 14–16, and 17–19 of the estrous cycle of Large White pigs. The protein expression was analyzed using western blot, and the results are presented as a densitometric normalized ratio relative to the β-actin abundance. The OMNT1 concentration in the culture medium was evaluated using ELISA. The results of at least four independent replicates are presented as means ± SEM for each group. Data were compared by an unpaired two-tailed Student’s t test (***p < 0.001, ****p < 0.0001). Representative blots are attached as Supplementary Fig. 1.

Furthermore, we observed that GnRH at 100 ng/mL for 24 h of cell culture increased OMNT1 protein expression during all phases of the estrous cycles, except on days 17–19, when we noted an inhibitory effect (*p* < 0.05; Fig. 5). Moreover, LH at 100 ng/mL significantly increased OMNT1 protein expression during all phases of the estrous cycle, while the effect of FSH at 100 ng/mL strongly depended on the day of the estrous cycle. We observed a stimulatory action on days 2–3 and 10–12 of the estrous cycle, no effect on days 14–16 of the estrous cycle, and an inhibitory effect on days 17–19 of the estrous cycle (*p* < 0.05; Fig. 5A–D). In addition, we observed no effect of GnRH, LH, and FSH on OMNT1 concentration in the culture medium (Figs. 4A′–C′ and 5A′–D′).

**Figure 6:**
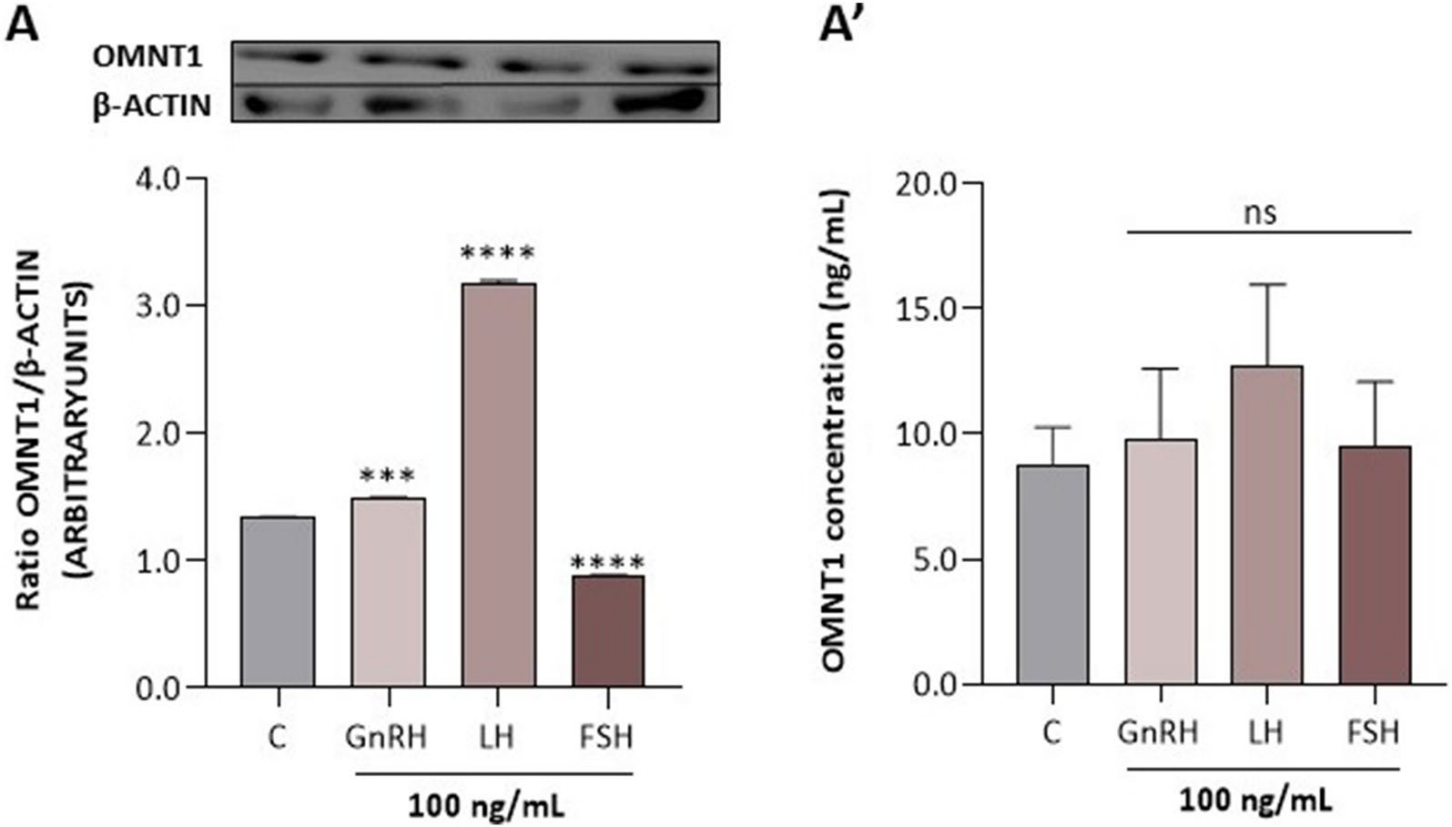
Effect of GnRH, LH, and FSH at 100 ng/mL on omentin-1 (OMNT1) protein expression (**A**) and levels in the culture medium (**A**′) of AP cells collected on days 10–12 of the estrous cycle of Meishan pigs. The protein expression was analyzed using western blot, and the results are presented as a densitometric normalized ratio relative to the β-actin abundance. The OMNT1 concentration in the culture medium was evaluated using ELISA. The results of at least four independent replicates are presented as means ± SEM for each group. Data were compared by an unpaired two-tailed Student's t test (***p < 0.001, ****p < 0.0001). Representative blots are attached as Supplementary Fig. 1.

Interestingly, we noted that GnRH and LH increased the protein expression of OMNT1 in pituitary cells on days 10–12 of the estrous cycle of MS pigs, while FSH had an inhibitory effect (*p* < 0.05; Fig. 6); however, no effect was observed on the concentration of OMNT1 in the culture medium.

## Discussion

To the best of our knowledge, this is the first study to show that plasma levels of OMNT1 and its expression in the AP gland fluctuated during the estrous cycle in two breeds of pigs. The obtained results also revealed an OMNT1 signal in the cytoplasm of somatotrophs, lactotrophs, thyrotrophs, and gonadotrophs in the porcine AP gland. Furthermore, GnRH and gonadotropins regulated OMNT1 protein expression in AP cells depending on the phase of the estrous cycle in LW pigs and had a modulatory effect in MS pigs.

OMNT1 was previously described in many tissues; however, data on OMNT1 expression in brain structures are scarce. Immunoreactivity of OMNT1 in neurons and glial cells in the human mesencephalon was observed^20^. Moreover, it may be involved in neuronal iron uptake and contribute to the degeneration of nigral dopaminergic neurons in Parkinson's disease^20,32^. OMNT1 may promote the growth and survival of neural stem cells in vitro^33^, ameliorate ischemic brain injury by promoting revascularization, and inhibit apoptosis in response to ischemia^32^, suggesting the potential use of OMNT1 in the treatment of vascular dementia caused by ischemic stroke and neurodegenerative diseases. In vivo studies by Brunetti et al.^14^ indicated modulatory effects of chronic OMNT1 on feeding behavior and related hypothalamic peptides and neurotransmitters in rats. Also, intraperitoneal injection of OMNT1 decreased CART and CRH gene expression and significantly increased norepinephrine synthesis and release^14^. However, there is currently limited data on the expression and physiology of OMNT1 in pituitary cells.

Here, we demonstrated that gene expression of *INTL1* increased with the progression of the estrous cycle in both breeds of pigs, with the highest transcript levels observed in AP of LW pigs from days 14–16 of the estrous cycle and in the gland of MS pigs from days 10–12 of the cycle. We observed the highest protein levels of OMNT1 on days 17–19 of the estrous cycle of LW and MS pigs. Differences in the patterns of INTL1 gene and protein levels may result from the complex regulation of transcriptional and post-transcriptional processes, differences in the stability of mRNA and proteins, and physiological feedback influencing transcript and protein concentrations^34^. Also, we observed a decrease in OMNT1 protein content in LW pigs on days 14–16 of the estrous cycle, when there are marked endocrine changes in P_4_ and E_2_ secretion and functional and structural luteolysis of the ovarian corpora lutea^35^. As is commonly known, tissue mRNA and protein levels are determined mainly by physiological states and are rarely correlated. Therefore, the changes in OMNT1 level that we observed in the AP gland during the estrous cycle can relate to the animal’s hormonal milieu, as was previously demonstrated for adiponectin^36^. The latest data also documented differences in chemerin expression in the porcine AP gland throughout the estrous cycle^37^. Interestingly, the adiponectin expression pattern in the rat pituitary gland was positively correlated with the P_4_ plasma level^36^. Also, Kieżun et al.^38^ described a higher adiponectin transcript level in the AP gland on days 2–3 of the estrous cycle, which may be due to the quick increase in P_4_ during that phase. Expression of other adipokines was described in the pituitary cells of human, rat, sheep, and pig brains^39^. For example, the *RARRES2* transcript has been found in the pituitary gland of baboons and chimpanzees^40^, and visfatin is present in mouse gonadotroph cells^41^. Based on our immunohistochemical analyses, OMNT1 protein was localized in the cytoplasm of somatotrophs, lactotrophs, thyrotrophs, and gonadotrophs of LW pigs which may suggest the influence of adipokine on their secretory functions. Similarly, nesfatin-1 was localized in endocrine cells in the AP gland of female mice and rats^42,43^, as well as in the cytoplasm of mouse LβT2 gonadotrophs^44^.

OMNT1 is known as a metabolic hormone whose levels fluctuate depending on the body’s energy state; previous reports indicate a negative correlation of OMNT1 levels with markers of obesity, such as the body mass index^5^. In adipose tissue, mRNA expression of *INTL1* was significantly lower in impaired glucose tolerance and type 2 diabetes mellitus patients than in control patients^45^. Data obtained by Barker et al.^28^ showed decreased transcript levels of *INTL1* in the placenta of maternal obesity patients compared with non-obese patients. Moreover, the presented results are in agreement with our previous study, which documented that plasma levels of OMN1 and its expression in peri-renal adipose tissue were significantly lower in fat MS pigs than in normal-weight LW pigs^18^. Interestingly, we observed a higher expression of OMNT1 in the AP gland collected from MS pigs than in that collected from LW pigs. These findings may reflect differences in metabolic hormones between the two breeds, such as increased E_2_ concentrations in MS pigs^46^ and FSH fluctuations^47^, which influence reproductive responses and fat mobilization. Thus, our results indicate that OMNT1 plasma levels and pituitary expression are strongly dependent on porcine metabolic status. Previously, a lower expression of vaspin was demonstrated in the ovarian follicles of LW pigs than in those of MS pigs^48^, and the opposite pattern of adiponectin expression was shown in the ovary of MS pigs compared with that of normal-weight Landrace pigs^49^. Various peptides or hormones found in peripheral cells, such as adipose visceral cells, could be biosynthesized in the pituitary gland and exert their physiological effects. Our results suggest that OMNT1 may be part of a brain system that operates independently, or in conjunction with, the peripheral systems. The evidence suggests that pituitary-derived adipokines represent a local regulatory circuit that may affect the feedback and control of female fertility and putative neuromodulators of energy balance. Literature data indicated that adiponectin increased basal FSH secretion by in vitro porcine AP cells in a dose- and phase-dependent manner during the estrous cycle^38^. Further research showed a decrease in basal and GnRH-stimulated LH secretion after short-time exposure to adiponectin in AP cells of male rats^50^. Studies on mouse LβT2 cells also confirmed the short-term inhibitory effect of adiponectin on LH secretion^51^. Moreover, chemerin has a modulatory effect on LH and FSH secretion in porcine AP cells; chemerin stimulated basal LH and FSH output on days 10–12 of the estrous cycle and inhibited LH secretion on days 14–16 and 17–19 of the estrous cycle^37^. However, more data are needed to understand the molecular mechanisms of the influence of OMNT1 on porcine pituitary function.

In contrast to our findings from the AP gland, we demonstrated higher blood levels of OMNT1 in LW pigs than in MS pigs, except on days 10–12 of the estrous cycle. Our results are in good agreement with data that demonstrated that circulating OMNT1 concentrations are negatively correlated with body fat in humans^25^. Also, plasma OMNT1 in humans was lower in obese people than in lean people^1,52^. We noted the highest plasma levels of OMNT1 on days 14–16 of the estrous cycle of LW pigs and the lowest levels on days 17–19 of the estrous cycle of MS pigs. Studies of healthy, normal-weight women showed stable plasma levels of OMNT1, with no associations between gonadotropins and E_2_ or P_4_ concentrations during the physiological menstrual cycle^53^. In our data, we also observed no correlation between plasma levels of OMNT1 and circulating FSH and LH levels in mature gilts regardless of body weight. Apart from the higher FSH and LH plasma concentrations observed in MS pigs than in LW pigs^47^, few endocrine differences exist between these breeds of females during the estrous cycle in the sow. The principal peaks in plasma FSH have also been shown to coincide with the preovulatory rise in LH^46^, which we confirmed in our study (Supplementary File 1). Literature data showed that the concentrations of vaspin and leptin increased in the mid-cycle and luteal phases compared with the follicular phase, proportional to changes in E_2_ and P_4_ levels. In contrast, plasma adiponectin levels showed an inverse trend during women’s physiological menstrual cycle^53^. Moreover, plasma resistin was positively correlated with LH and LH/FSH^54^. Furthermore, taking into account different OMNT1 expressions in the pituitary gland and plasma, it appears that OMNT1 may function through autocrine mechanisms in AP cells. In our study, we found that circulating LH and FSH levels were positively correlated with OMNT1 abundance in AP, suggesting that both gonadotropins may stimulate pituitary OMNT1 protein expression*.* Artimani et al.^55^ observed a strong positive correlation between the expression of the adiponectin receptors AdipoR1 and AdipoR2 in granulosa cells and the expression of FSH and LH receptors. Other data indicated that the expression of leptin receptors in Leydig cells was inversely correlated with serum testosterone concentration^56^. The pituitary gland is not protected by the blood–brain barrier and is chronically bathed in blood-borne substances; for example, Jéquier et al.^57^ suggested that pituitary-derived leptin might “tune” the leptin-signaling pathways to incoming leptin signals from adipose tissue. Although OMNT1’s ability to cross the blood–brain barrier is still unknown, one cannot exclude the effect of both local pituitary-produced and systemic-produced hormones on reproductive functions. Previous data have shown that serum and cerebrospinal fluid (CSF) levels of resistin are elevated in human and animal models of obesity and diabetes, implicating dysregulation of resistin in these diseases^58^. Also, circulating adiponectin enters the brain fluid from the circulation, and the trimer and hexamer forms of adiponectin can be detected in the CSF^59^.

Based on our findings that indicated differences in OMNT1 expression during the estrous cycle, we focused on determining factors that can regulate OMNT1 levels in AP cells. Results of our in vitro study clearly showed that OMNT1 expression increased in response to GnRH and gonadotropins at all doses used on days 10–12 of the estrous cycle of LW pigs, but modulatory effects (stimulatory or inhibitory) were observed depending on the phase of the estrous cycle. However, we observed that GnRH, LH, and FSH have no effect on OMNT1 levels in the culture medium of AP cells; the difference between protein expression of OMNT1 and its concentration in culture medium can be explain the sensitivity and specificity of the tests used. As is commonly known, western blot generate more information about the target protein and the sample than ELISA, moreover, it’s less likely to give false-positive results, as it can effectively distinguish between different antibodies, which is confirmed by numerous studies. To detect various proteins involved in the occurrence of the nervous system diseases neurocysticercosis and Trichinella, western blot analysis had almost 100% sensitivity and specificity^60,61^. Interactions between the nervous and endocrine systems require a complex communication network composed of many hormones, including OMNT1.

It is well known that the production and secretion of gonadotropins are regulated by the frequency of GnRH pulses, which play an important role in the development of sex function and the normal regulation of the menstrual or estrous cycle. Disturbance of neuroendocrine regulation and GnRH pulsatility in the hypothalamus causes a reduction in sex hormones, an absence of or delay in sexual maturation, and a reduced competence of gametes manifested by abnormal ovulation, contributing to the complex etiology of female infertility^62^. Our results showed that GnRH has stimulatory effects on the regulation of OMNT1 expression in pituitary cells, except for 100 ng/mL on days 17–19 of the estrous cycle in LW pigs. To the best of our knowledge, this is the first study to demonstrate a regulatory effect of GnRH on OMNT1 expression. However, GnRH significantly decreased mRNA expression of adiponectin in both LβT2 gonadotrophs and rat primary pituitary cells^36^. On the other hand, an inhibitory role of adiponectin in GnRH neuron activity was noted in the hypothalamus^63^. Moreover, irisin enhanced the transcript level of GnRH and increased the release of GnRH by hypothalamic GT1-7 cells^64^.

The gonadotropins released from the pituitary gland in response to GnRH are involved in female reproduction through the regulation of ovaries' function. FSH regulates the growth and maturation of follicles and stimulates the production of estrogens, whereas LH plays a primary role in ovulation and initiates the luteinization of follicular cells. Moreover, in pigs, cows, and sheep, LH is necessary for the normal development of the corpus luteum and the maintenance of its function^65^. In this study, we noted that LH had a stimulatory effect on the pituitary expression of OMNT1 at the doses used (50–150 ng/mL) during the estrous cycle in LW and MS pigs; thus, this effect was not dependent on dose, the phase of the estrous cycle, or the metabolic status of the animal. Our research showed the LH levels fluctuated approximately 50 ng/mL in the pre-ovulatory surge, and FSH levels increased as the cycle progressed (approximately 150 ng/mL; Supplementary Fig. 2). The use of a dose of 100 ng/ml of gonadotropins had a more successful effect on OMNT1 levels in AP cells. However, we observed that the effect of FSH was dependent on the estrous cycle in LW pigs: a stimulatory action on days 2–3 and 10–12 of the estrous cycle, no effect on days 14–16 of the estrous cycle, and an inhibitory effect on days 17–19 of the estrous cycle. Data on the regulation of OMNT1 levels by gonadotropins are very limited and only indicated that FSH at a dose of 10 nM decreased the expression of OMNT1 in granulosa cells^10^. However, previous studies have reported that gonadotropins are involved in the regulation of leptin^66^, adiponectin and its receptors^67,68^, apelin and its receptor^69^, resistin^70^, and vaspin^48^ in ovarian cells. Treatment of rats with FSH and LH to induce follicular development and ovulation increased the expression of AdipoR1^71^. We also examined the effect of these hormones on OMNT1 regulation in the mid-luteal phase of MS pigs. FSH significantly reduced OMNT1 expression in MS pigs compared with LW pigs, suggesting that differences in OMNT1 levels may be related to differences in the hormonal profile of these breeds. Literature data showed higher levels of inhibin in MS pigs than in LW pigs, resulting in reduced FSH secretion from the AP gland^47^. Moreover, higher levels of gonadotropin release were noted in LW pigs than in MW pigs^72^, which may explain the stimulating effect of GnRH, LH, and FSH, especially in the mid-luteal phase. Other factors, produced by central or peripheral tissue, may also take part in this regulation. For example, MS pigs are characterized by an elevated concentration of cortisol, which can increase LH levels^47^.

Taken together, the present study has indicated that plasma OMNT1 levels and OMNT1 expression in the porcine AP gland depend on the phase of the estrous cycle and the hormonal status of the animal. Also, our in vitro results indicated that the levels of OMNT1 increased in response to LH during the estrous cycle, while the effects of FSH and GnRH strongly depended on the phase of the estrous cycle and the breed of pig. These data may also serve as a basis for future studies on how hormonal regulation in the female porcine pituitary gland depends on the status of the animal’s energy metabolism. Further studies are required to examine the role of OMNT1 in the autoregulation function of AP cells, including the secretion of tropic hormones, especially gonadotropins, and the mechanisms underlying the described processes.

## Methods

### Animals and collection of samples

Animals were killed and tissues were collected as a by-product according to the European Communities Council Directive 2010/63/EU on the protection of animals used for scientific purposes, adopted on September 22, 2010. Therefore, in accordance with the animal protection rules, the studies submitted did not require the approval of the relevant ethics committee for experiments on animals. Samples were collected from pigs intended for slaughter for research, commercial purposes, and meat processing (research unit of the National Research Institute for Agriculture, Food and the Environment, Nouzilly, France; slaughter and breeding farm, Poland). Mature normal-weight LW pigs (91.76 ± 8.2 kg) and fat MS pigs (30.62 ± 5.8 kg) were used in this study. In our previous study, we observed that MS animals had a higher backfat thickness (BFT) than LW animals^18^; also, all the zootechnical parameters (body weight, backfat thickness, age at the puberty) were routinely collected by the UEPAO (doi: https://doi.org//10.15454/1.5573896321728955E12) and Pig phenotyping and Innovative breeding facility (https://doi.org//10.15454/1.5572415481185847E12) experimental units for the monitoring of the breeding. Blood and pituitary glands were collected from pigs in particular phases of the estrous cycle, which were assessed on the basis of ovarian morphology^73^ and plasma profile of E_2_ and P_4_^74^. In order to study OMNT1 expression, LW and MS females were divided into four experimental groups according to the four phases of the estrous cycle (n = 6 per group), as follows: days 2–3 (early luteal phase, presence of the corpora hemorrhagica), days 10–12 (mid-luteal phase, fully functional corpora lutea), days 14–16 (late luteal phase, regression corpora), and days 17–19 (follicular phase) of the estrous cycle (Supplementary Fig. 2). Within a few minutes after slaughter, blood samples were collected into heparinized tubes and then centrifuged (2000×*g* for 15 min at 4 °C), and plasma was stored at − 20 °C until further measurement. Pituitary glands were placed in phosphate-buffered saline (PBS, Thermo Fisher Scientific, USA) supplemented with 100 IU/mL penicillin (Sigma-Aldrich, St. Louis, MO, USA) and 100 µg/mL streptomycin (Sigma-Aldrich, St. Louis, MO, USA) and transported to the laboratory on ice. The pituitary glands were dissected and divided into AP and posterior lobes, and further research was conducted only on AP lobes. The anterior lobes were frozen in liquid nitrogen and stored at − 70 °C until processing for mRNA and protein expression. For immunohistochemical determinations, the AP gland was isolated on days 10–12 of the estrous cycle of LW pigs (n = 3), which was placed in 4% buffered paraformaldehyde (pH = 7.4, 4 °C) for immunohistochemistry.

### Reverse transcription quantitative real-time polymerase chain reaction (RT-qPCR)

RNA from the pituitary gland was extracted with QIAzol lysis reagent (cat. no. 79306, Qiagen, Germany) according to the manufacturer's instructions. Concentration and purity (260/280) of isolated RNA were determined with a NanoDrop spectrophotometer (Peqlab Biotechnologie GmbH, Erlangen, Germany). To obtain cDNA, reverse transcription (RT) of total RNA (1 μg) was performed (for 60 min at 37 °C, total volume 20 μl). The reaction mixture contained deoxynucleotide triphosphates, 10 × reverse transcription buffer, oligodeoxythymidylic acid, recombinant ribonuclease inhibitor, and Moloney murine leukemia virus reverse transcriptase (cat. no. U1515, A3561, C1101, N2515, and M1705, respectively, Promega, USA). Specific primers for *ITLN1* and the reference genes *PPIA* (cyclophilin A), *ACTB* (β-actin), and *GAPDH* (glyceraldehyde-3-phosphate dehydrogenase), which were not affected by the breed or the time of the estrous cycle, were used for qPCR; the primers were designed based on the gene sequences deposited in GenBank. The reaction mixture contained 10 μl iQ SYBR Green Supermix (cat. no. 1708885, Bio-Rad, USA), 0.25 μl of each primer (10 μM), 4.5 μl of RNAse-free water, and 5.0 μl of cDNA (the total volume of 20 μl contained 20 ng of cDNA). Templates were amplified in the presence of specific primers, *ITLN1* (forward 5′-GATTCTGCCTCCTGCTGTTC-3′ and reverse 5′-ATACAGGCCATCACCTGCTC-3′), *PPIA* (forward 5′-GCATACAGGTCCTGGCATCT-3′ and reverse 5′-TGTCCACATGCAGC AATGGT-3′), *ACTB* (forward 5′-ACGGAACCACAGTTTATCATC-3′ and reverse 5′-GTCCCAGT CTTCAACTATACC-3′), and *GAPDH* (forward 5′-GCACCGTCAAGGCTGAGAAC-3′ and reverse 5′-ATGGTGGTGAAGACGCCAGT-3′), using the device MyiQ Cycle (Bio-Rad, France). After the samples were incubated for 2 min at 50 °C with a denaturation step of 10 min at 95 °C, the samples were subjected to 40 cycles (30 s at 95 °C, 30 s at 60 °C, 30 s at 72 °C), followed by the acquisition of the melting curve; the samples were amplified on the same plate as previously described^18^. Relative gene expressions were determined using the comparative cycle threshold 2 − ΔΔCt method^75^. The relative expression of the target gene was then normalized by the geometric mean of the expression levels of the reference gene. Negative controls contained RNAse-free water instead of cDNA.

### Western blot

Lysates from the whole AP gland and cultured AP cell were obtained using lysis buffer (50 mM Tris, HCl pH 7.6, 150 mM NaCl, 1% NP-40, 0.5% sodium deoxycholate, 0.1% SDS) with a cocktail of protease inhibitors (cat. no. 200-664-3, Sigma-Aldrich, St. Louis, MO, USA). The lysates were centrifuged (10,000×*g* for 10 min at 4 °C), the supernatant was collected, and the protein concentration was measured by the bicinchoninic acid protein assay (cat. no. 23225, Thermo Fisher Scientific, USA). Protein lysates (20 μg protein/lane), with the addition of Laemmli (cat. no. 38733, Sigma-Aldrich, St. Louis, MO, USA) after denaturation (95 °C for 5 min), were loaded on hand-casting 10% sodium dodecyl sulfate–polyacrylamide gels in an electrophoresis chamber. Membranes were transferred to polyvinylidene fluoride membranes (cat. no. IPVH00010, Sigma-Aldrich, St. Louis, MO, USA) and blocked (for 60 min, 25 °C) with Tris-Buffered Saline Tween (TBST) buffer, containing 0.1% of Tween 20 and 5% of bovine serum albumin (BSA, cat. no. ALB001.500, BioShop, Canada). The membranes were incubated overnight at 4 °C with anti-OMNT1 antibody diluted 1:500 (cat. no. sc-130923, Santa Cruz Biotechnology, USA). The membranes were rinsed with TBST buffer and incubated for 60 min with anti-mouse antibodies conjugated with horseradish peroxidase diluted 1:1000 (cat. no. 7074S, Cell Signaling Technology, USA). Anti-β-actin protein was used as loading control (diluted 1:1000; cat. no. A5316, Sigma-Aldrich, St. Louis, MO, USA), which was detected on the same membranes as the target protein. Proteins of interest were detected by chemiluminescence using Immobilon Western Chemiluminescent HRP Substrate (cat. no. WBKLS0500, Millipore, USA) visualized using the ChemiDoc™ imaging system (BioRad). Then, the proteins were quantified using densitometric analysis and ImageJ software (US National Institutes of Health, USA).

### Enzyme-linked immunosorbent assay (ELISA)

The blood samples were used to determine the level of OMNT1 and its correlation with the gonadotropins FSH and LH in plasma. In addition, the level of OMNT1 in the culture medium was determined. According to the manufacturer's protocol, the measurements were performed using commercially available porcine ELISA kits: OMNT1 (cat. no. E07O0010, BlueGene Biotech, China), FSH (cat. no. EP0060, FineTest, China), LH (cat. no. EP0105, FineTest, China), E_2_ (cat. no. EIA-2693, DRG Diagnostics, Germany), and P_4_ (cat. no. EIA-1561, DRG Diagnostics, Germany). The intra‐assay coefficient of variation of the assay was 10% for OMNT1, 8% for FSH, 10% for LH, 9.2% for E_2,_ and 6.86% for P_4_. For all measurements, absorbance values were measured at 450 nm using a Varioskan™ LUX multimode microplate reader (Thermo Fisher Scientific, USA). Standard curves were plotted using Curve Expert software (Hyams Development, USA). Curve fitting was confirmed by the value of the coefficient of determination, which amounted to > 0.99 for all analyses.

### Immunofluorescence

The AP glands collected on days 10–12 of the estrous cycle of LW pigs were incubated in 4% paraformaldehyde for 48 h and rinsed under running water for 24 h. The AP lobes were incubated in ethanol (successively 50%, 70%, 96%, and 100%; each series for 48 h) and absolute ethanol with xylene (50:50) for 5 h and twice with xylene alone for 9 h. Tissues were supersaturated with paraffin (melting point 56–58 °C) at least for 24 h at 60 °C, embedded in paraffin blocks, and cut into slices. Eosin and hematoxylin staining was performed to confirm tissue morphology. For immunofluorescence staining, the paraffin slices were incubated for 2 h at 60 °C and twice in xylene for 15 min. The slices were incubated in ethanol (100–50% for 10 min) and acetone (for 20 min at − 20 °C), boiled in Antigen Unmasking Solution Tris-Based (500–600 V microwave for 3 × 5 min; cat. no. H-3301, Vector Laboratories, UK), incubated in 50 mM NH_4_CL (for 30 min) and 0.1% Triton X-100 (for 10 min), and blocked in Fish Serum Blocking Buffer (for 90 min; cat. no. 37527, Thermo Fisher Scientific, USA). After each incubation with the reagents, the slices were rinsed in PBS for 15 min. Finally, the sections were incubated overnight with rabbit polyclonal antibodies against OMNT1 diluted 1:100 (PA5-96614, Thermo Fisher Scientific, USA) and mixed with mouse monoclonal antisera against tropic hormones: GH diluted 1:200 (ab218405, Abcam, UK), PRL diluted 1:200 (cat. no. ab11301, Abcam, UK), ACTH diluted 1:500 (cat. no. ab212736, Abcam, UK), TSH diluted 1:200 (cat. no. MAB57941, R&D, USA), LH diluted 1:200 (cat. no. ab212578, Abcam, UK), and FSH diluted 1:300 (cat. no. ab233866, Abcam, UK); then the sections were washed three times with PBS for 15 min. Next, the sections were incubated for 90 min with a mixture solution composed of Alexa Fluor 488 anti-rabbit antibodies diluted 1:1000 (cat. no. A-11008, Thermo Fisher Scientific, USA) and Alexa Fluor 594 anti-mouse antibodies diluted 1:1000 (cat. no. 115–585-003, Jackson ImmunoResearch, UK). After staining, the slices were rinsed and incubated with 0.5% Sudan Black B in 70% EtOH for 20 min, and then the glasses were air-dried for 5–10 min. The sections were covered with coverslipping glasses with a mounting medium containing 4′,6-diamidyno-2-fenyloindol (Sigma-Aldrich, St. Louis, MO, USA) for nuclear counterstaining. The labeled AP sections were analyzed with an Olympus BX51 research microscope (Olympus, Japan) equipped with an EXFO x‐Cite Series 120Q fluorescence illuminator (Excelitas Technologies Corp) using appropriate filters. Images were acquired with an Olympus DP72 microscope digital camera and Cell F software (Olympus, Japan). Negative controls were performed via the omission and replacement of primary antisera by non-immunosera to test antibody and method specificity. The lack of any immunoreactions indicated specificity.

### In vitro cultures of AP cells

To study the effect of GnRH, FSH, and LH on OMNT1 expression and secretion, in vitro cultures of AP cells were made based on the protocol described by Kieżun et al. (2014) (n = 4 at least) with some modifications. For in vitro experiments, APs were pooled in order to obtain a sufficient number of cells. The AP gland was isolated and cut into 1–2 mm fragments and isolated in Dulbecco’s modified Eagle’s medium (DMEM, Sigma-Aldrich, St. Louis, MO, USA) with 0.1% BSA supplemented with 100 IU/mL penicillin and 100 µg/mL streptomycin. Single-cell suspensions were obtained by several digestions with 0.2% collagenase type V (cat. no. C9263, Sigma-Aldrich, St. Louis, MO, USA) and/or with 0.25% pancreatin (cat. no. P1750, Sigma-Aldrich, St. Louis, MO, USA) in DMEM for 10 min at 37 °C. After several centrifugations (1200 rpm for 10 min), dispersed pituitary cells were passed through a nylon filter (70 μm mesh) to remove undigested tissue fragments. Cell viability (90–97%) was determined with 0.4% trypan blue (cat. no. 15250061, Thermo Fisher Scientific, USA) and counted using a Bürker chamber. Cells were cultured in McCoy's 5A medium containing 10% horse serum (cat. no. H1270, Sigma-Aldrich, St. Louis, MO, USA) with a 1% mixture of antibiotics (100 U/mL penicillin, 100 ug/mL streptomycin, 2.5 ug/mL amphotericin B; cat. no. 15240062, Thermo Fisher Scientific, USA) supplemented with 0.1% minimum essential medium (MEM) vitamins (cat. no. 11120052, Thermo Fisher Scientific, USA) and 0.01% MEM nonessential amino acids (cat. no. 11140050, Thermo Fisher Scientific, USA). Cell viability (90–97%) was determined by trypan blue dye exclusion. The AP cell cultures, in 96-well culture plates at a density of 10 × 10^4^ cells per well, were preincubated for 72 h at 37 °C under a water-saturated atmosphere of 5% CO_2_ and 95% air. Then, the AP cells were cultured in McCoy's 5A medium with 1% horse serum or hormones in three experimental groups for 24 h (Supplementary Fig. 3) In the first experimental group, we tested the dose-dependent effect. AP cells were isolated on days 10–12 of the estrous cycle (phase chosen based on OMNT1 expression results) of LW pigs and treated with GnRH (cat. no. L8008, Sigma-Aldrich, St. Louis, MO, USA), LH (cat. no. L5259, Sigma-Aldrich, St. Louis, MO, USA), and FSH (cat. no. F4021, Sigma-Aldrich, St. Louis, MO, USA) at doses of 50, 100, and 150 ng/mL. The hormone doses were chosen based on our preliminary data and the literature^38^. In the second experimental group, we tested the estrous cycle-dependent effect. AP cells were isolated on days 2–3, 10–12, 14–16, and 17–19 of the estrous cycle of LW pigs, after which the cells were treated with GnRH, LH, and FSH at a dose of 100 ng/mL, which was chosen on the basis of previous dose-dependent experiments. In the third experimental group, we tested the effect of GnRH, LH, and FSH in MS pigs. AP cells were isolated on days 10–12 of the estrous cycle and treated with GnRH, LH, and FSH at a dose of 100 ng/mL (cycle phase and hormone concentration were chosen on the basis of previous analyses on LW pigs). After 24 h of incubation, the culture medium was collected and centrifuged at 1000×*g*, while the cells were stored at − 20 °C for ELISA and western blot analysis.

### Statistical analyses

All experimental data are presented as means ± standard error of the mean (SEM) from at least four independent experiments. The distribution of normality was checked with a Shapiro–Wilk test, and the homogeneity of variances with Levene’s test. Statistical analysis was carried out using a two-way ANOVA, followed by Tukey’s test (GraphPad software) to compare OMNT1 levels between breeds of pigs (LW and MS) and days of the estrous cycle. Furthermore, a two-tailed Student’s t test was used to compare the effect of GnRH, LH, and FSH with the control group. Correlations between plasma concentrations of OMNT1 and those of the gonadotropins FSH, LH, E_2_, and P_4_ were analyzed with the Pearson correlation coefficient (two-tailed *p* < 0.05). Statistical significance is indicated by different letters (*p* < 0.05) or by ***p < 0.001 and ****p < 0.0001.

### Ethics declarations and approvals for animal experiments

Blood and pituitary glands were a by-product from animals intended for research or commercial purposes (meat processing). This study did not require the approval of the ethics committee for experiments on animals, because the slaughter of animals, the collection of biological material, and the transport of material to the laboratory were carried out in accordance with the Polish Act on the Protection of Animals Used for Scientific or Educational Purposes of January 15, 2015 (Journal of Laws Dz.U. 2015 No. item 266) and the European Communities Council Directive 2010/63/UE of September 22, 2010, on the protection of animals used for scientific purposes.

### Supplementary Information

Supplementary Figure S1.

Supplementary Figure S2.

Supplementary Figure S3.

## Data Availability

The datasets used and/or analysed during the current study available from the corresponding author on reasonable request.
