# Supplementary material for: Plasma level of omentin-1, its expression, and its regulation by gonadotropin-releasing hormone and gonadotropins in porcine anterior pituitary cells
Source: Sci Rep. 2023 Nov 7;13:19325. doi: 10.1038/s41598-023-46742-4 (PMC10630491; doi:10.1038/s41598-023-46742-4)

Plasma level of omentin-1, its expression, and its regulation by gonadotropin-releasing hormone and gonadotropins in porcine anterior pituitary cells

Natalia Respekta, Karolina Pich, Ewa Mlyczyńska, Kamil Dobrzyń, Christelle Ramé, Joëlle Dupont, Tadeusz Kamiński, Nina Smolińska, Agnieszka Rak

Representative original blots to protein expression of omentin-1 in the anterior pituitary gland collected on days 2-3, 10-12, 14-16, and 17-19 of the estrous cycle of Large White (LW) and Meishan (MS) pigs (Fig. 1B).

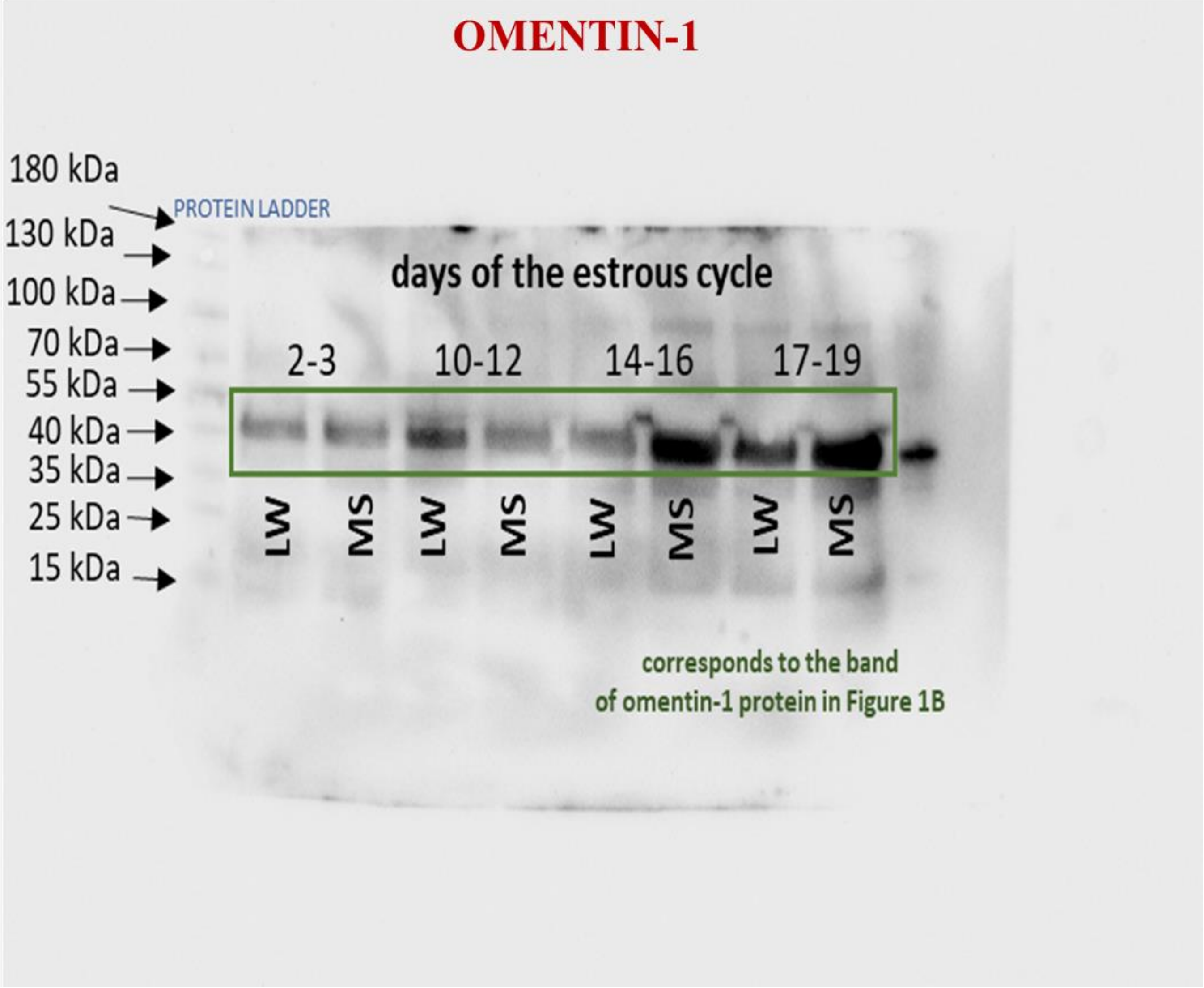

Plasma level of omentin-1, its expression, and its regulation by gonadotropin-releasing hormone and gonadotropins in porcine anterior pituitary cells

Natalia Respekta, Karolina Pich, Ewa Mlyczyńska, Kamil Dobrzyń, Christelle Ramé, Joëlle Dupont, Tadeusz Kamiński, Nina Smolińska, Agnieszka Rak

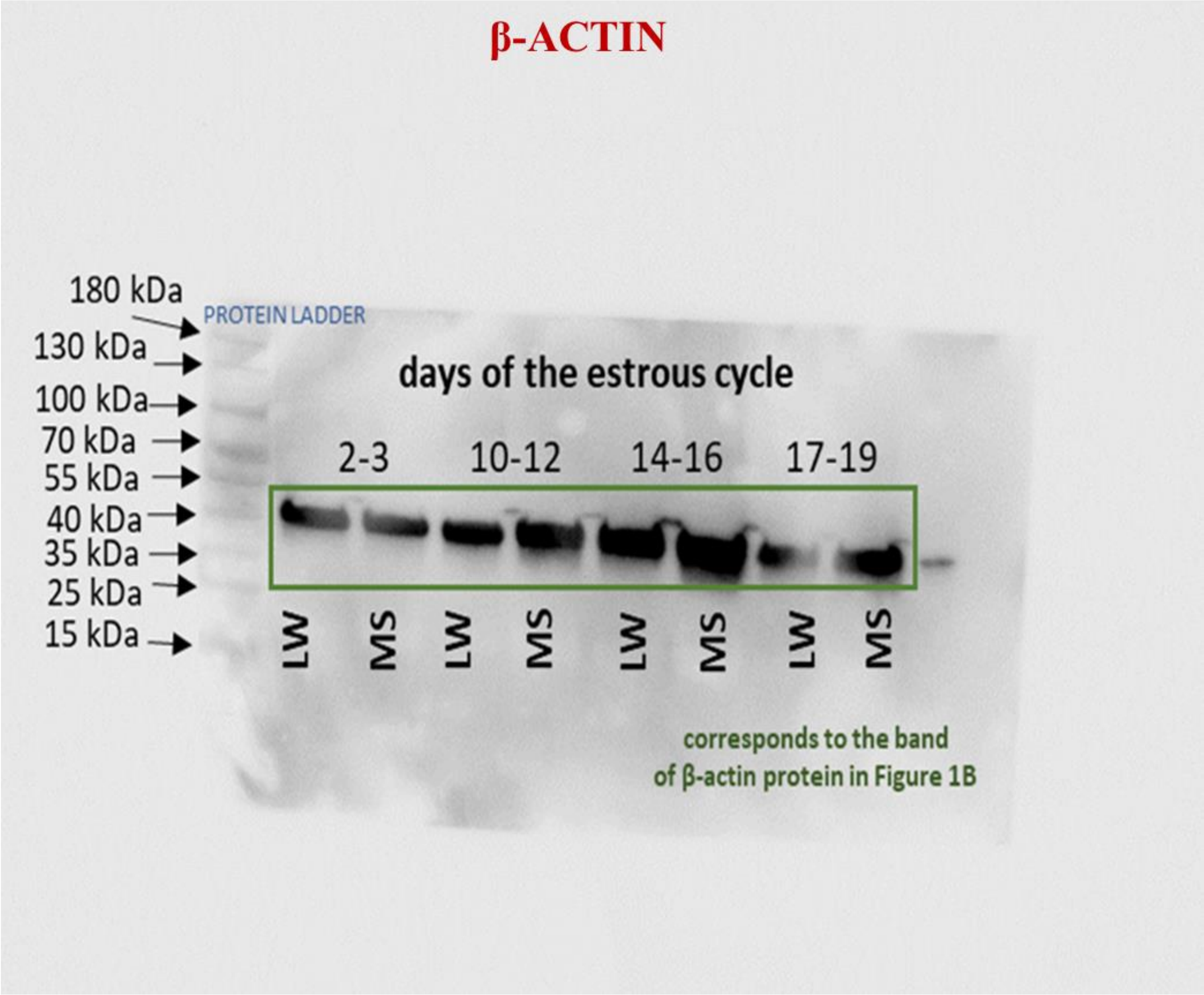

Plasma level of omentin-1, its expression, and its regulation by gonadotropin-releasing hormone and gonadotropins in porcine anterior pituitary cells

Natalia Respekta, Karolina Pich, Ewa Mlyczyńska, Kamil Dobrzyń, Christelle Ramé, Joëlle Dupont, Tadeusz Kamiński, Nina Smolińska, Agnieszka Rak

Representative original blots to impact of GnRH, LH, and FSH on omentin-1 protein expression in anterior pituitary cells depending on the doses (50-150 ng/ml) and phase of the estrous cycle (days 2-3, 10-12, 14-16, and 17-19) of LW and MS pigs (Fig. 4A-C, 5A-D, 6A).

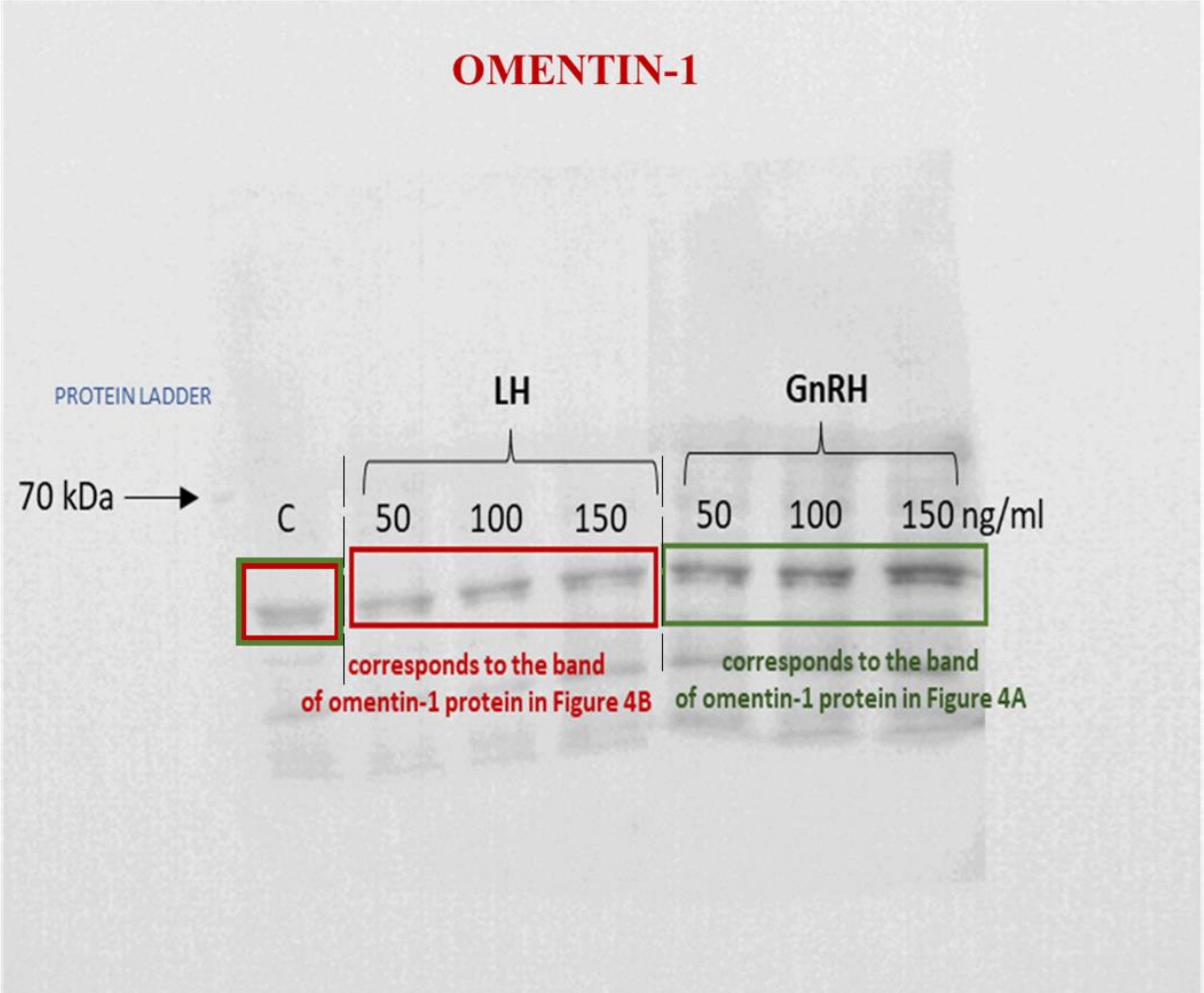

Plasma level of omentin-1, its expression, and its regulation by gonadotropin-releasing hormone and gonadotropins in porcine anterior pituitary cells

Natalia Respekta, Karolina Pich, Ewa Mlyczyńska, Kamil Dobrzyń, Christelle Ramé, Joëlle Dupont, Tadeusz Kamiński, Nina Smolińska, Agnieszka Rak

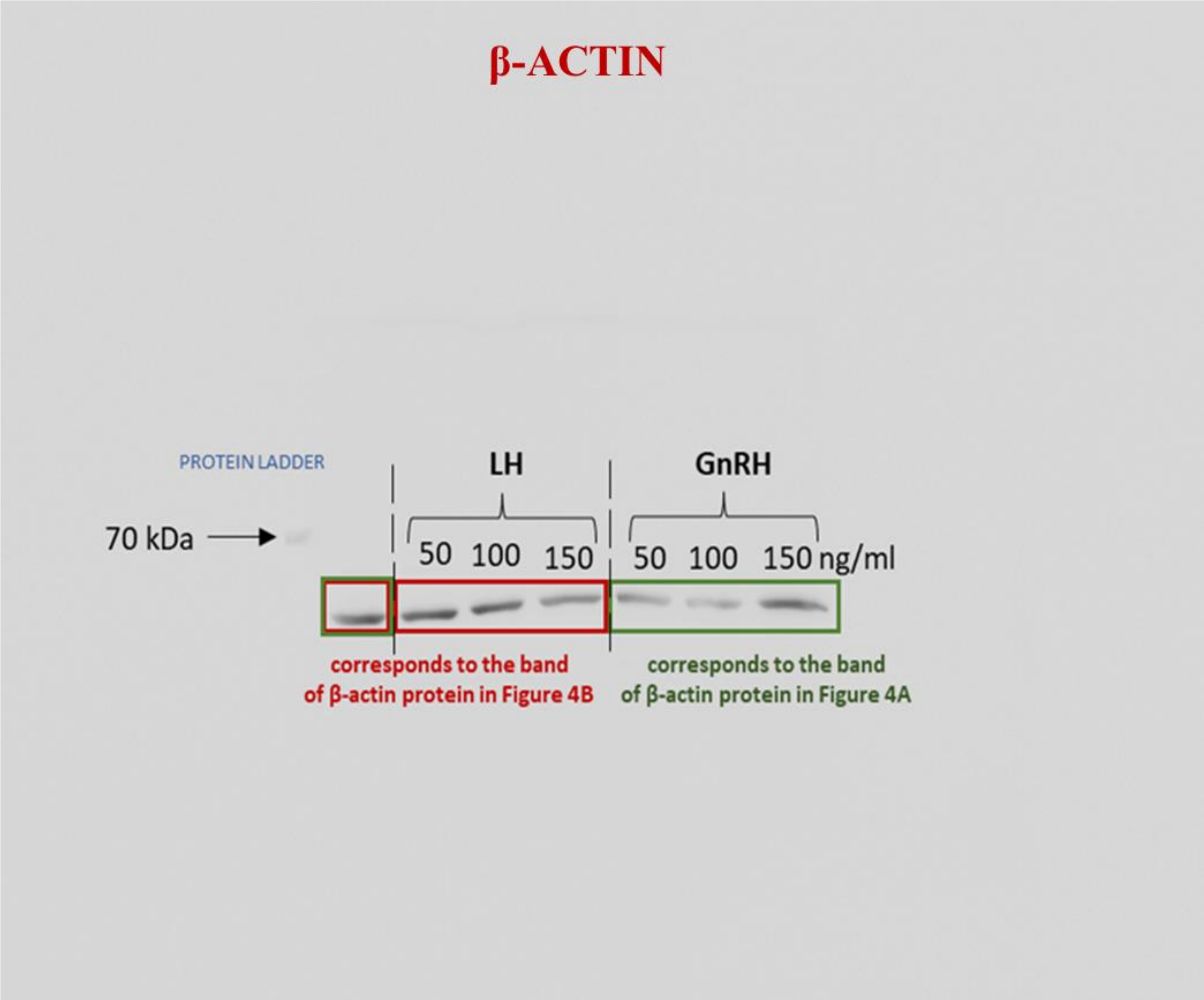

Plasma level of omentin-1, its expression, and its regulation by gonadotropin-releasing hormone and gonadotropins in porcine anterior pituitary cells

Natalia Respekta, Karolina Pich, Ewa Mlyczyńska, Kamil Dobrzyń, Christelle Ramé, Joëlle Dupont, Tadeusz Kamiński, Nina Smolińska, Agnieszka Rak

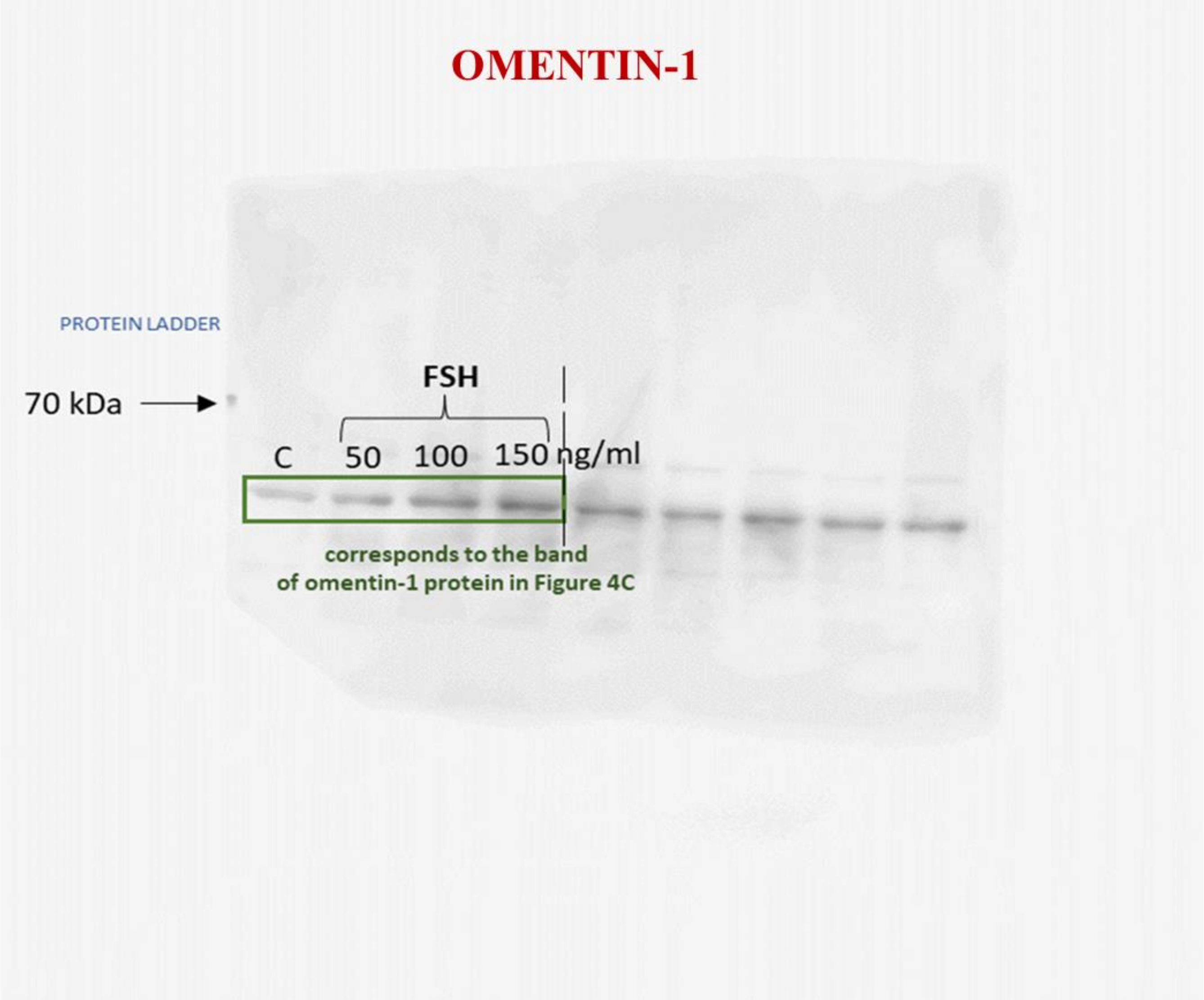

Plasma level of omentin-1, its expression, and its regulation by gonadotropin-releasing hormone and gonadotropins in porcine anterior pituitary cells

Natalia Respekta, Karolina Pich, Ewa Mlyczyńska, Kamil Dobrzyń, Christelle Ramé, Joëlle Dupont, Tadeusz Kamiński, Nina Smolińska, Agnieszka Rak

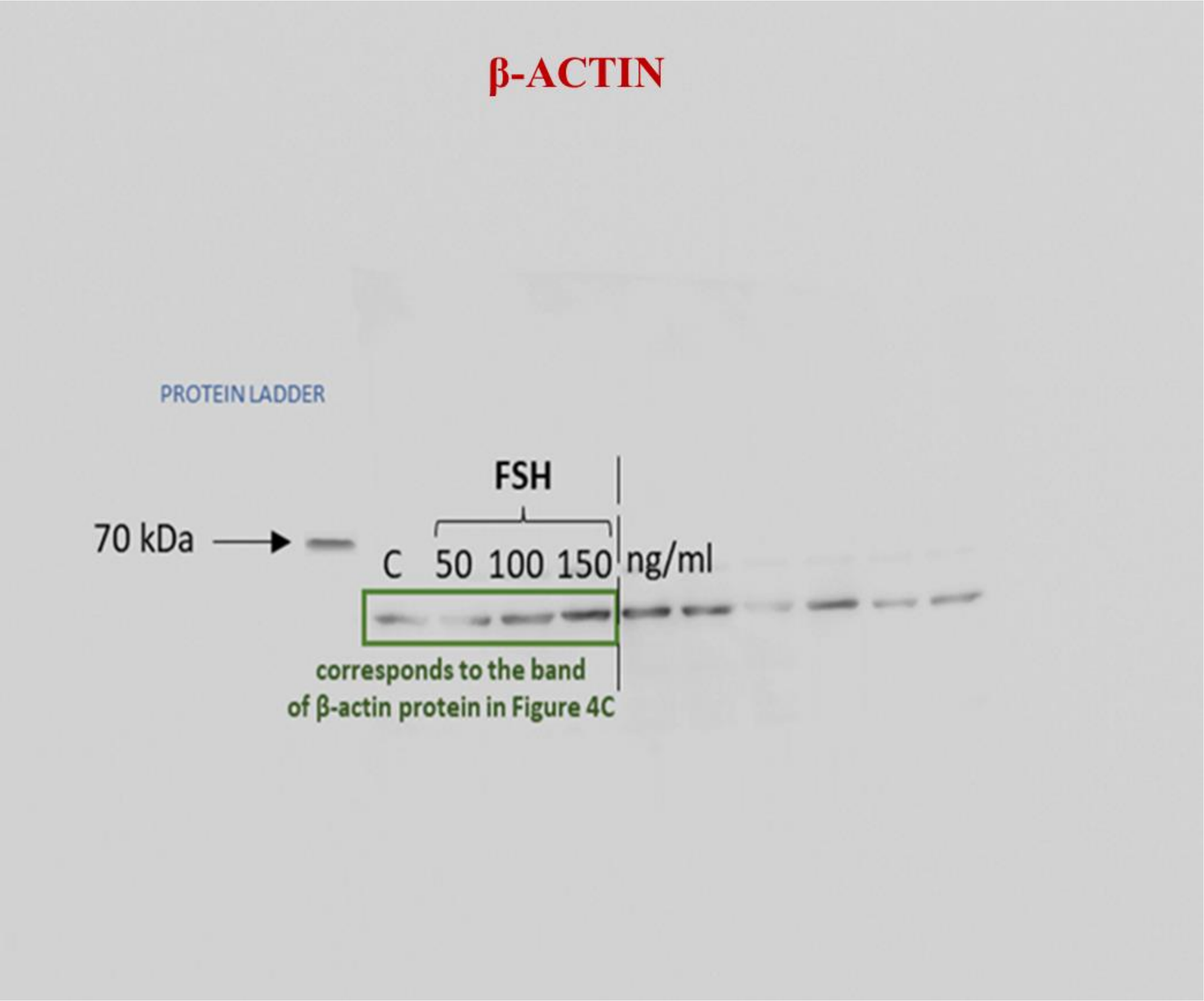

Plasma level of omentin-1, its expression, and its regulation by gonadotropin-releasing hormone and gonadotropins in porcine anterior pituitary cells

Natalia Respekta, Karolina Pich, Ewa Mlyczyńska, Kamil Dobrzyń, Christelle Ramé, Joëlle Dupont, Tadeusz Kamiński, Nina Smolińska, Agnieszka Rak

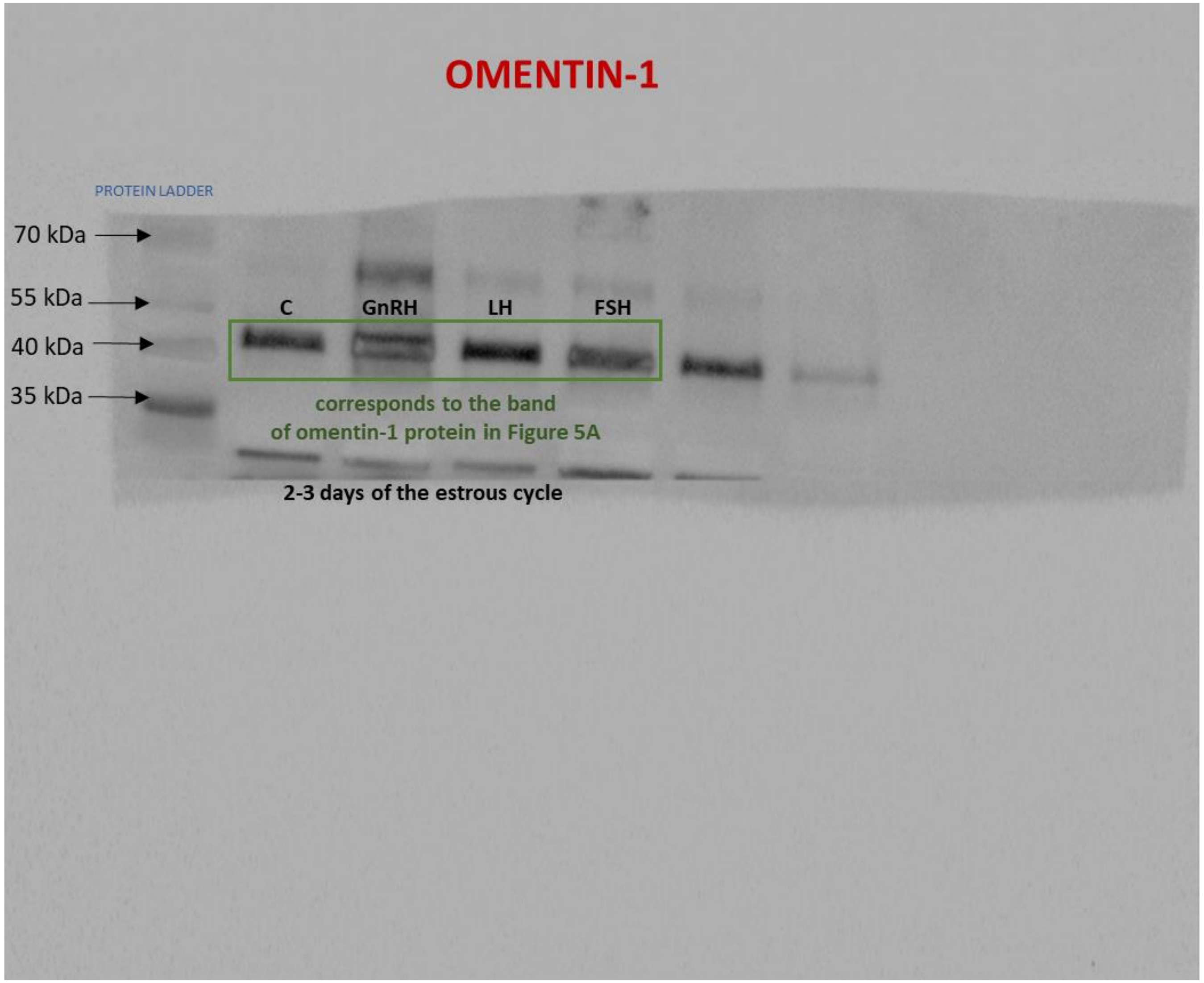

*the membrane was cut horizontally for another experiment

Plasma level of omentin-1, its expression, and its regulation by gonadotropin-releasing hormone and gonadotropins in porcine anterior pituitary cells

Natalia Respekta, Karolina Pich, Ewa Mlyczyńska, Kamil Dobrzyń, Christelle Ramé, Joëlle Dupont, Tadeusz Kamiński, Nina Smolińska, Agnieszka Rak

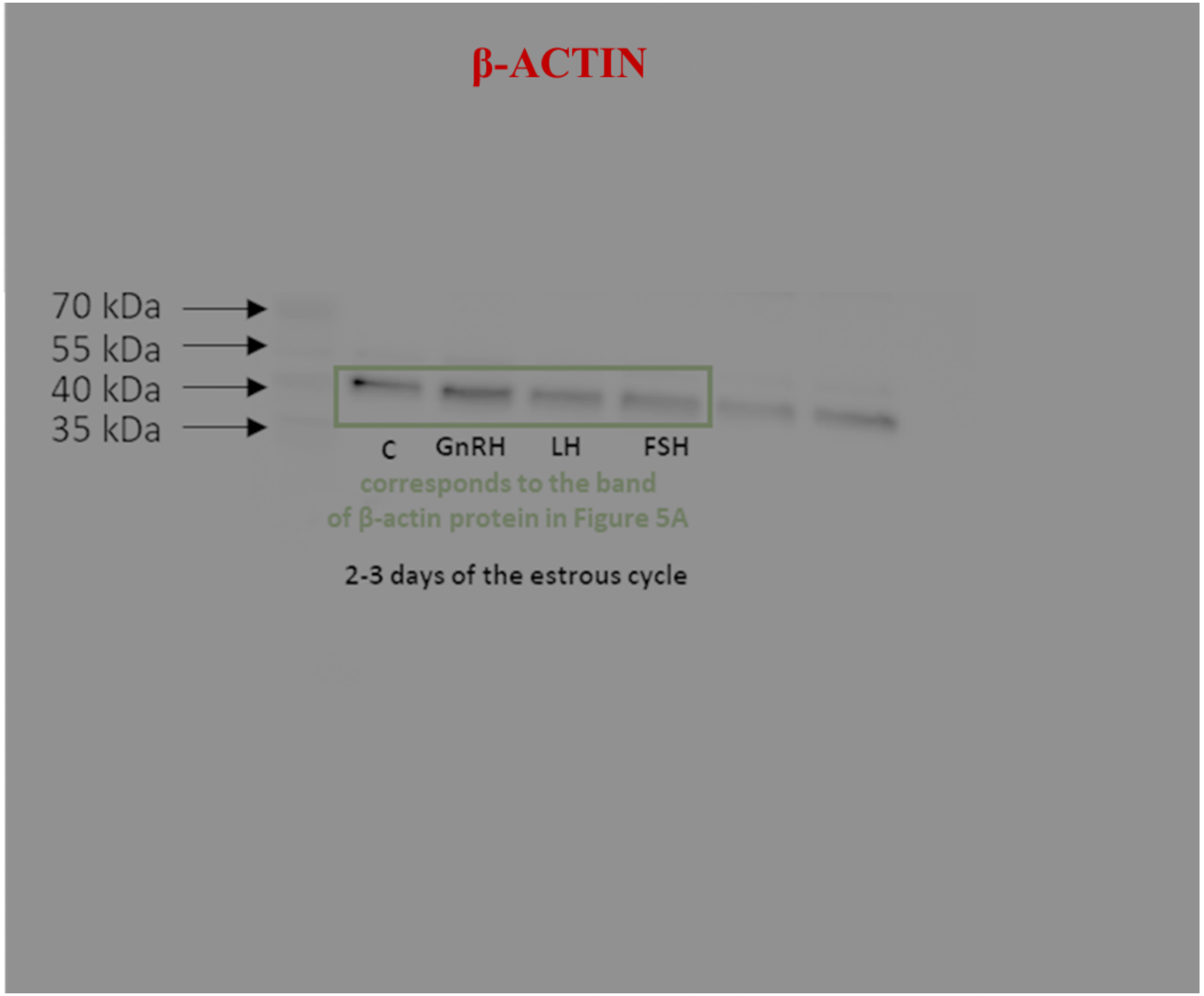

*the membrane was cut horizontally for another experiment

Plasma level of omentin-1, its expression, and its regulation by gonadotropin-releasing hormone and gonadotropins in porcine anterior pituitary cells

Natalia Respekta, Karolina Pich, Ewa Mlyczyńska, Kamil Dobrzyń, Christelle Ramé, Joëlle Dupont, Tadeusz Kamiński, Nina Smolińska, Agnieszka Rak

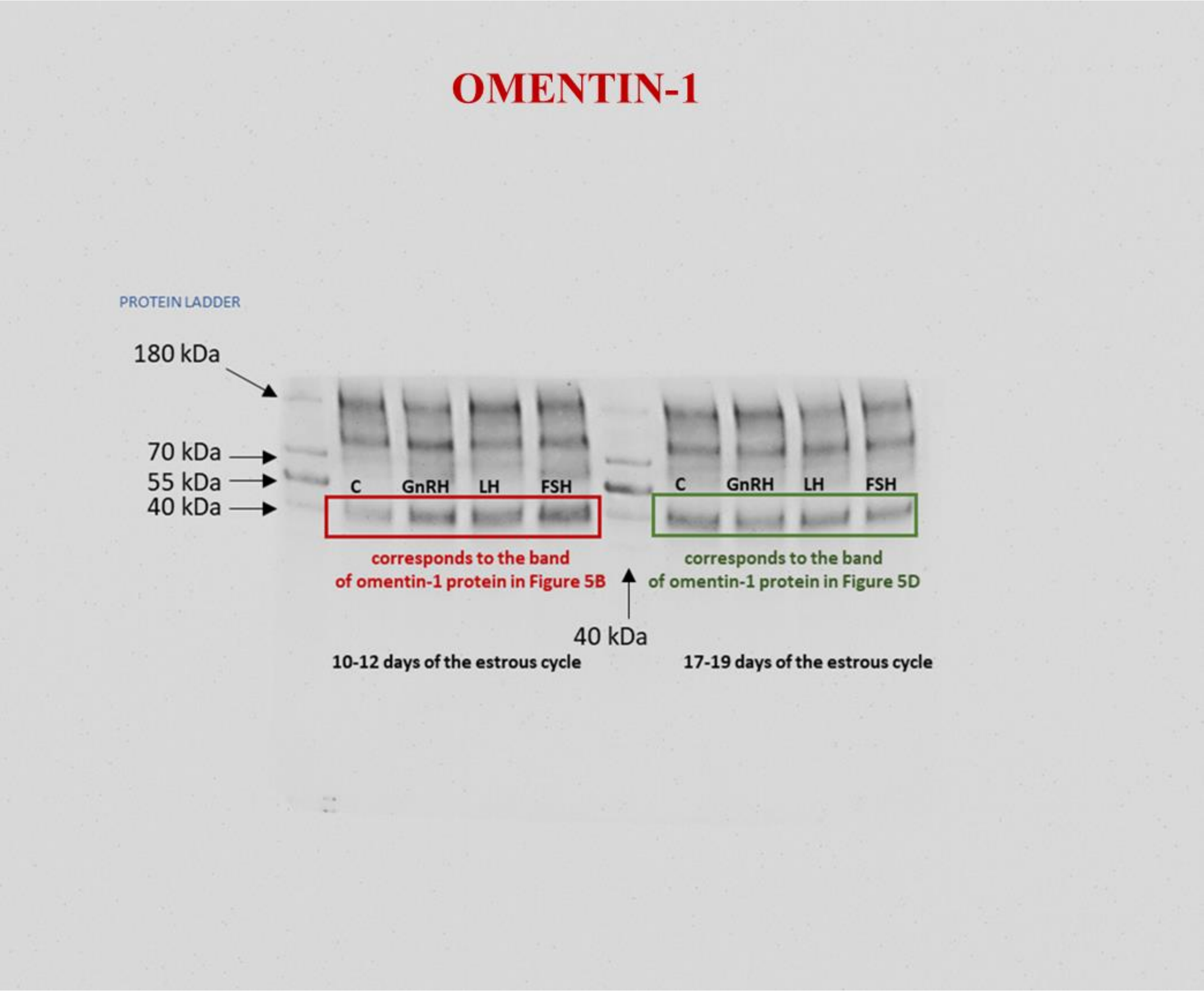

Plasma level of omentin-1, its expression, and its regulation by gonadotropin-releasing hormone and gonadotropins in porcine anterior pituitary cells

Natalia Respekta, Karolina Pich, Ewa Mlyczyńska, Kamil Dobrzyń, Christelle Ramé, Joëlle Dupont, Tadeusz Kamiński, Nina Smolińska, Agnieszka Rak

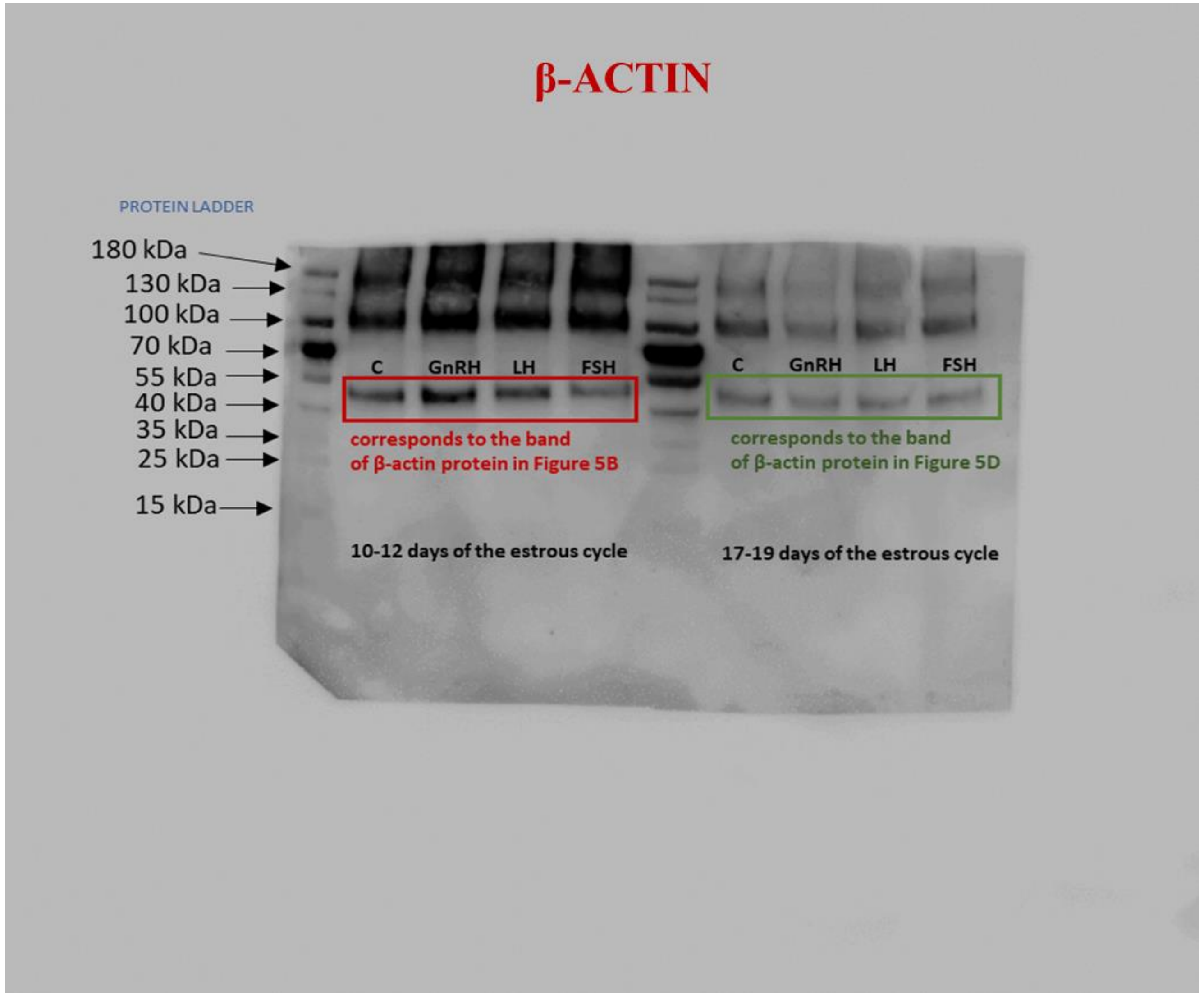

Plasma level of omentin-1, its expression, and its regulation by gonadotropin-releasing hormone and gonadotropins in porcine anterior pituitary cells

Natalia Respekta, Karolina Pich, Ewa Mlyczyńska, Kamil Dobrzyń, Christelle Ramé, Joëlle Dupont, Tadeusz Kamiński, Nina Smolińska, Agnieszka Rak

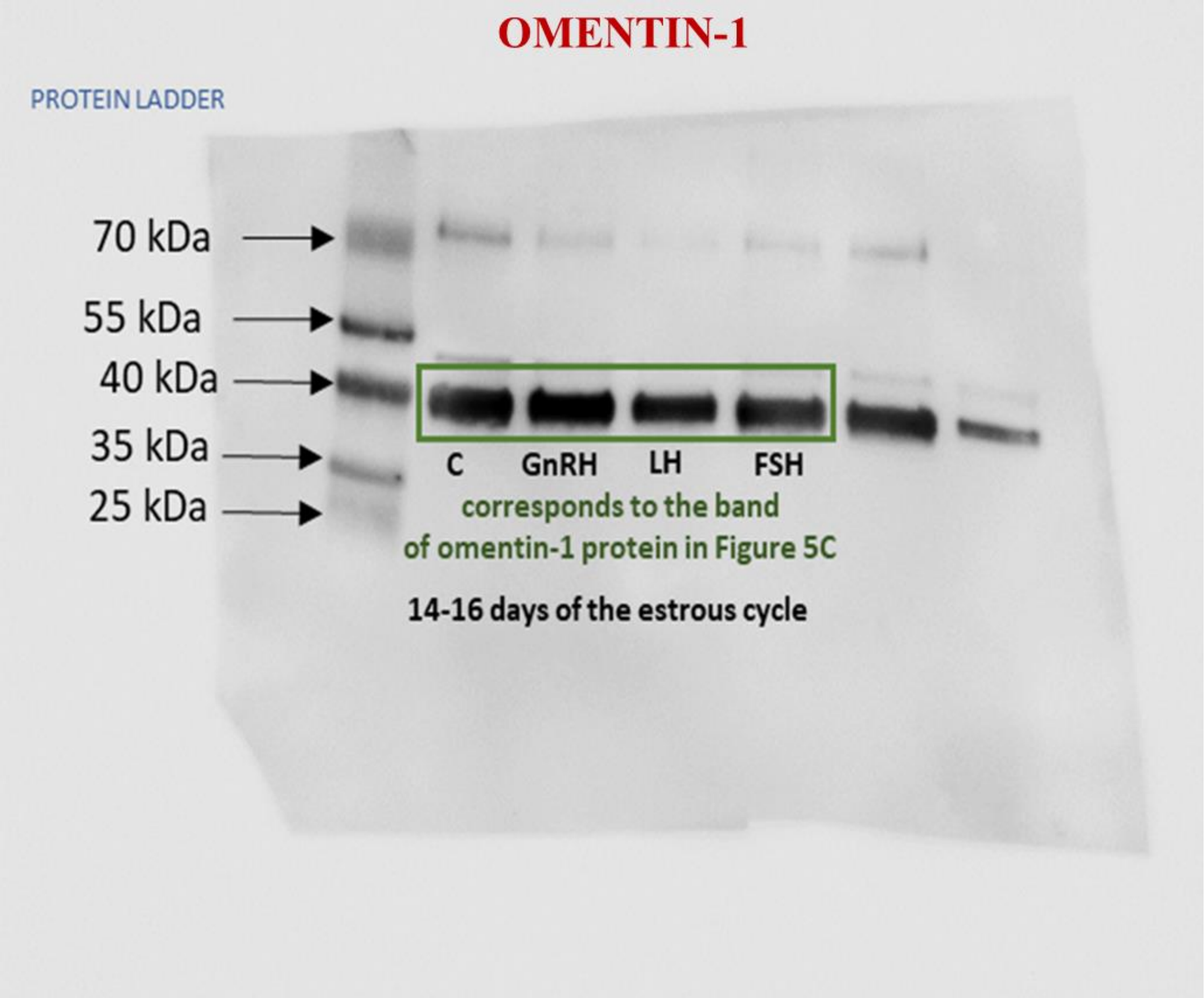

Plasma level of omentin-1, its expression, and its regulation by gonadotropin-releasing hormone and gonadotropins in porcine anterior pituitary cells

Natalia Respekta, Karolina Pich, Ewa Mlyczyńska, Kamil Dobrzyń, Christelle Ramé, Joëlle Dupont, Tadeusz Kamiński, Nina Smolińska, Agnieszka Rak

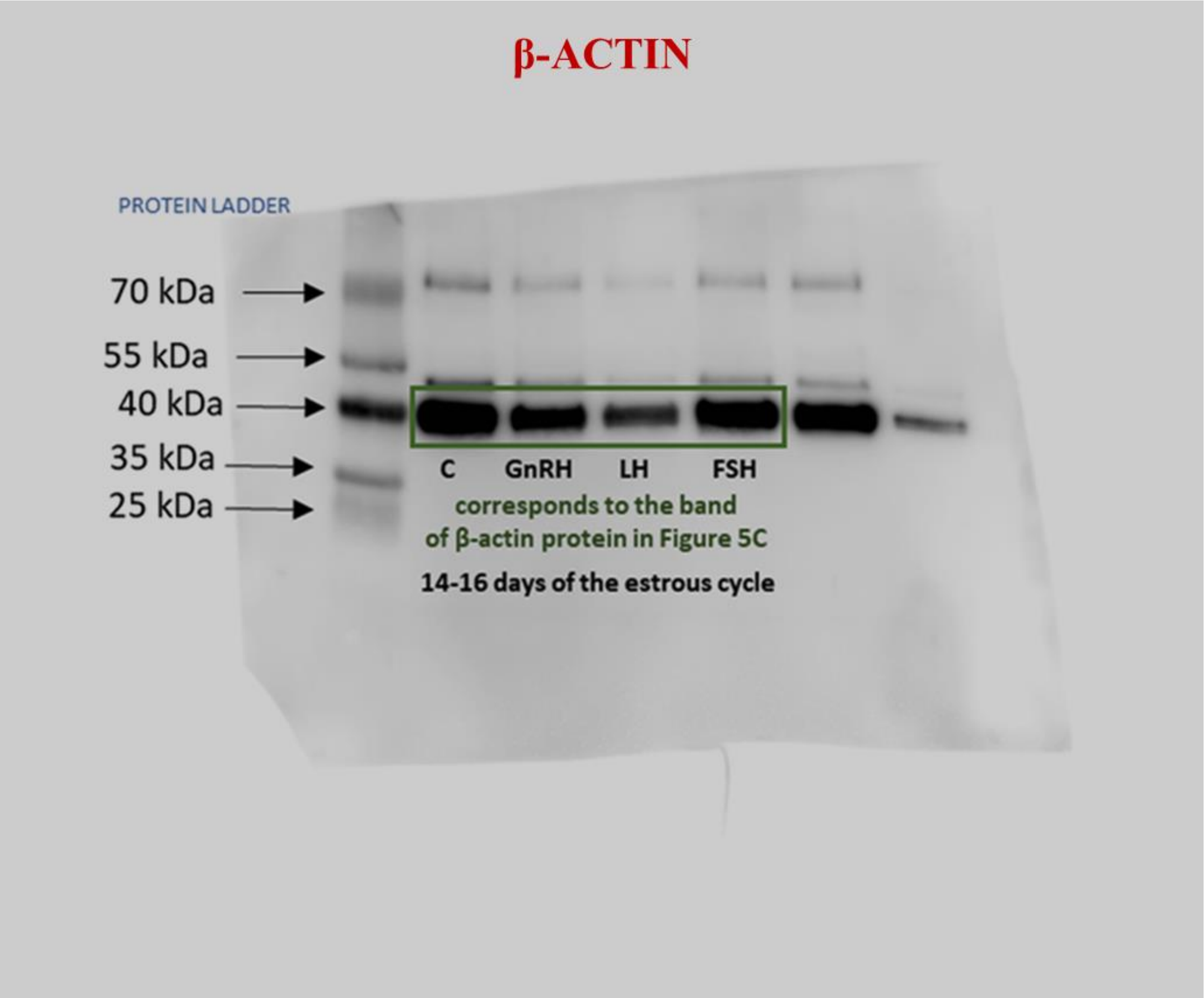

Plasma level of omentin-1, its expression, and its regulation by gonadotropin-releasing hormone and gonadotropins in porcine anterior pituitary cells

Natalia Respekta, Karolina Pich, Ewa Mlyczyńska, Kamil Dobrzyń, Christelle Ramé, Joëlle Dupont, Tadeusz Kamiński, Nina Smolińska, Agnieszka Rak

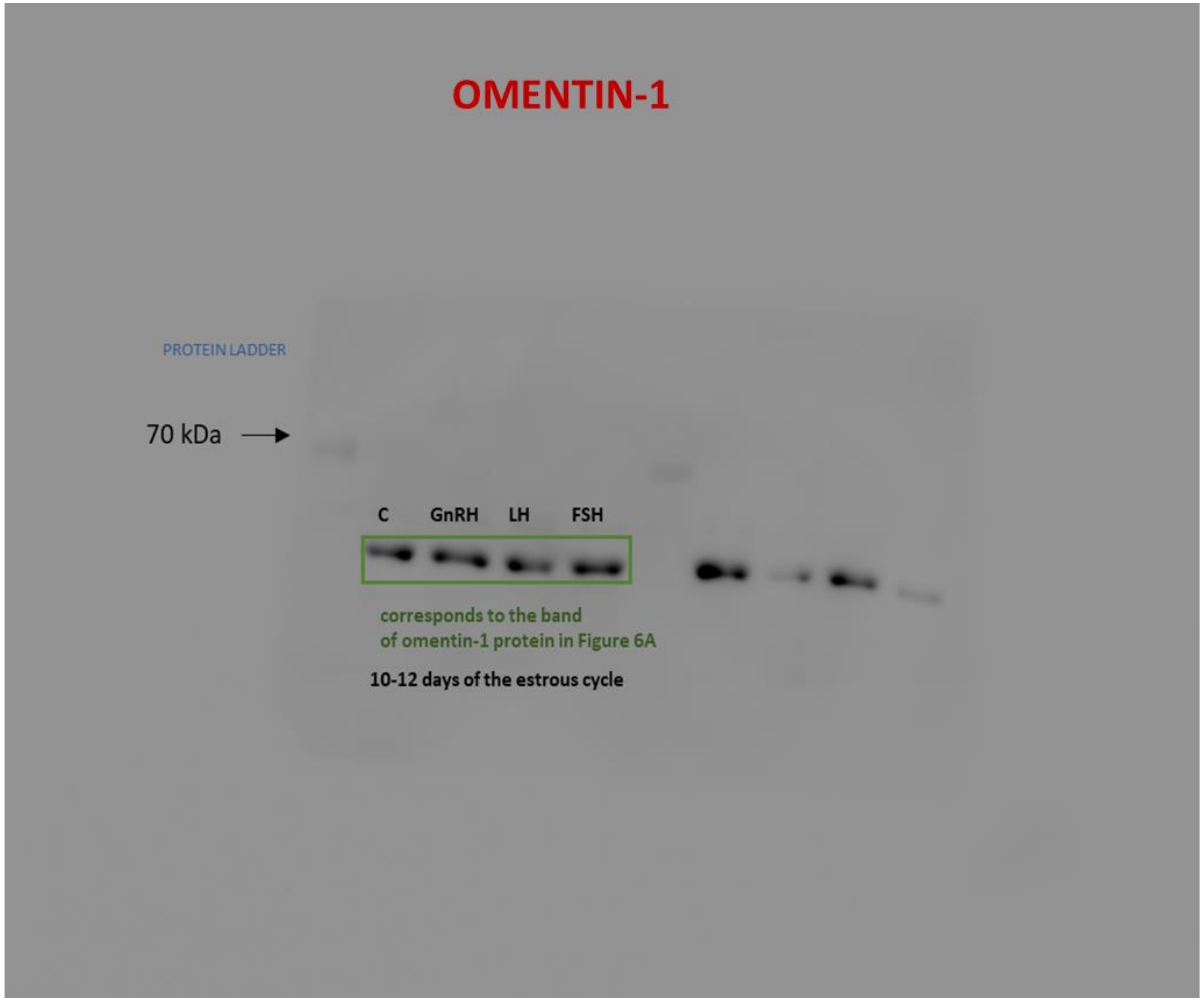

Plasma level of omentin-1, its expression, and its regulation by gonadotropin-releasing hormone and gonadotropins in porcine anterior pituitary cells

Natalia Respekta, Karolina Pich, Ewa Mlyczyńska, Kamil Dobrzyń, Christelle Ramé, Joëlle Dupont, Tadeusz Kamiński, Nina Smolińska, Agnieszka Rak

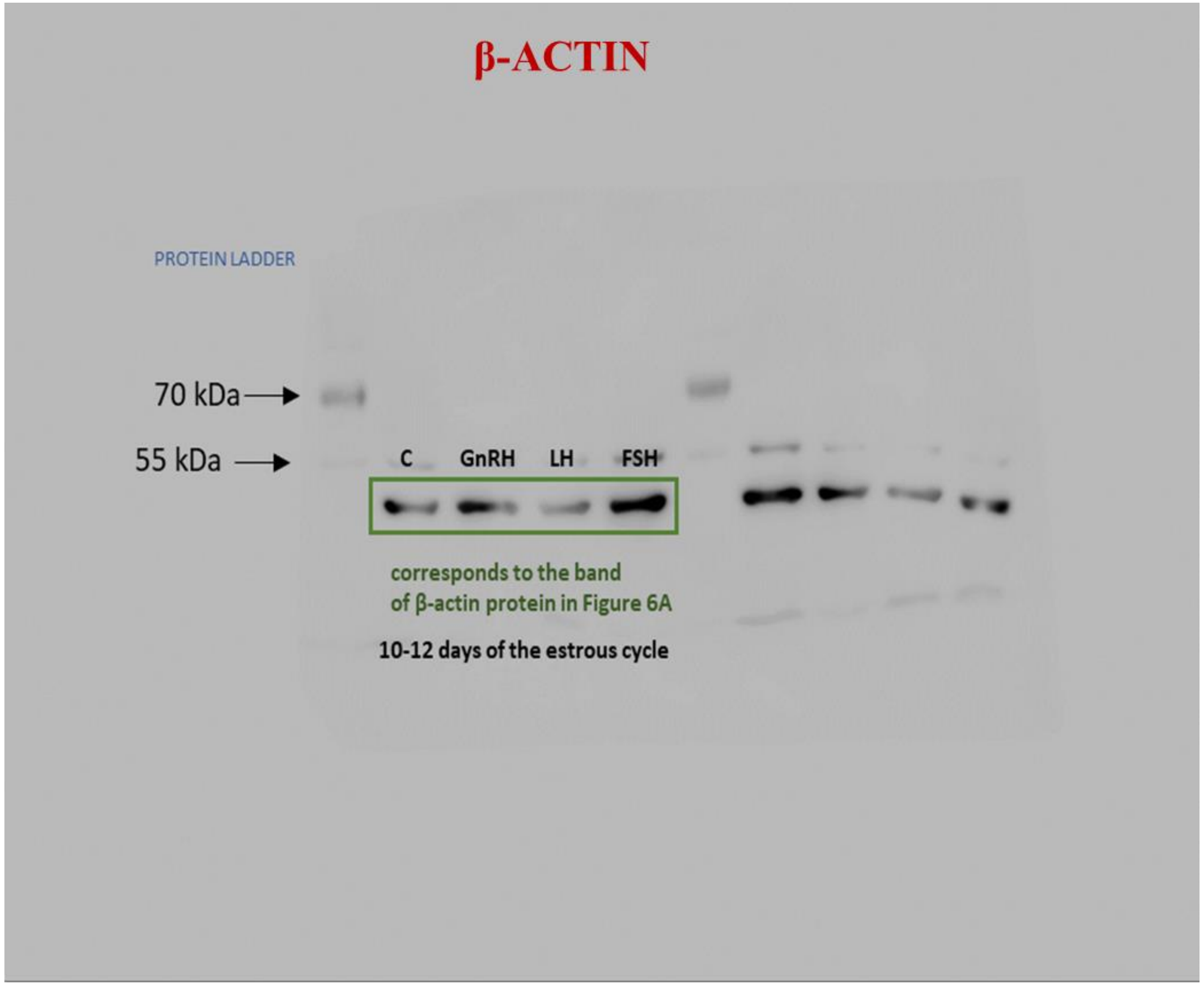

Supplement: Supplementary file 1 — Supplementary Figure S1. [file 41598_2023_46742_MOESM1_ESM.pdf]
