# Supplementary material for: Plasma level of omentin-1, its expression, and its regulation by gonadotropin-releasing hormone and gonadotropins in porcine anterior pituitary cells
Source: Sci Rep. 2023 Nov 7;13:19325. doi: 10.1038/s41598-023-46742-4 (PMC10630491; doi:10.1038/s41598-023-46742-4)
Supplement: Supplementary file 2 — Supplementary Figure S2. [file 41598_2023_46742_MOESM2_ESM.docx]

**Plasma level of omentin-1, its expression, and its regulation by** **gonadotropin-releasing hormone and gonadotropins in porcine anterior pituitary cells**

Natalia Respekta, Karolina Pich, Ewa Mlyczyńska, Kamil Dobrzyń, Christelle Rame, Joëlle Dupont, Tadeusz Kamiński, Nina Smolińska, Agnieszka Rak

Plasma concentration of gonadotropins (LH and FSH) and steroids (P_4_ and E_2_) on days 2-3, 10-12, 14-16, and 17-19 of the estrous cycle of Large White and Meishan pigs. The hormone concentrations were evaluated using ELISA. Results are presented as at least six independent replicates as means ± SEM for each group. Bars with different superscripts are significantly different (p < 0.05).
