# Supplementary material for: Plasma level of omentin-1, its expression, and its regulation by gonadotropin-releasing hormone and gonadotropins in porcine anterior pituitary cells
Source: Sci Rep. 2023 Nov 7;13:19325. doi: 10.1038/s41598-023-46742-4 (PMC10630491; doi:10.1038/s41598-023-46742-4)
Supplement: Supplementary file 3 — Supplementary Figure S3. [file 41598_2023_46742_MOESM3_ESM.pdf]

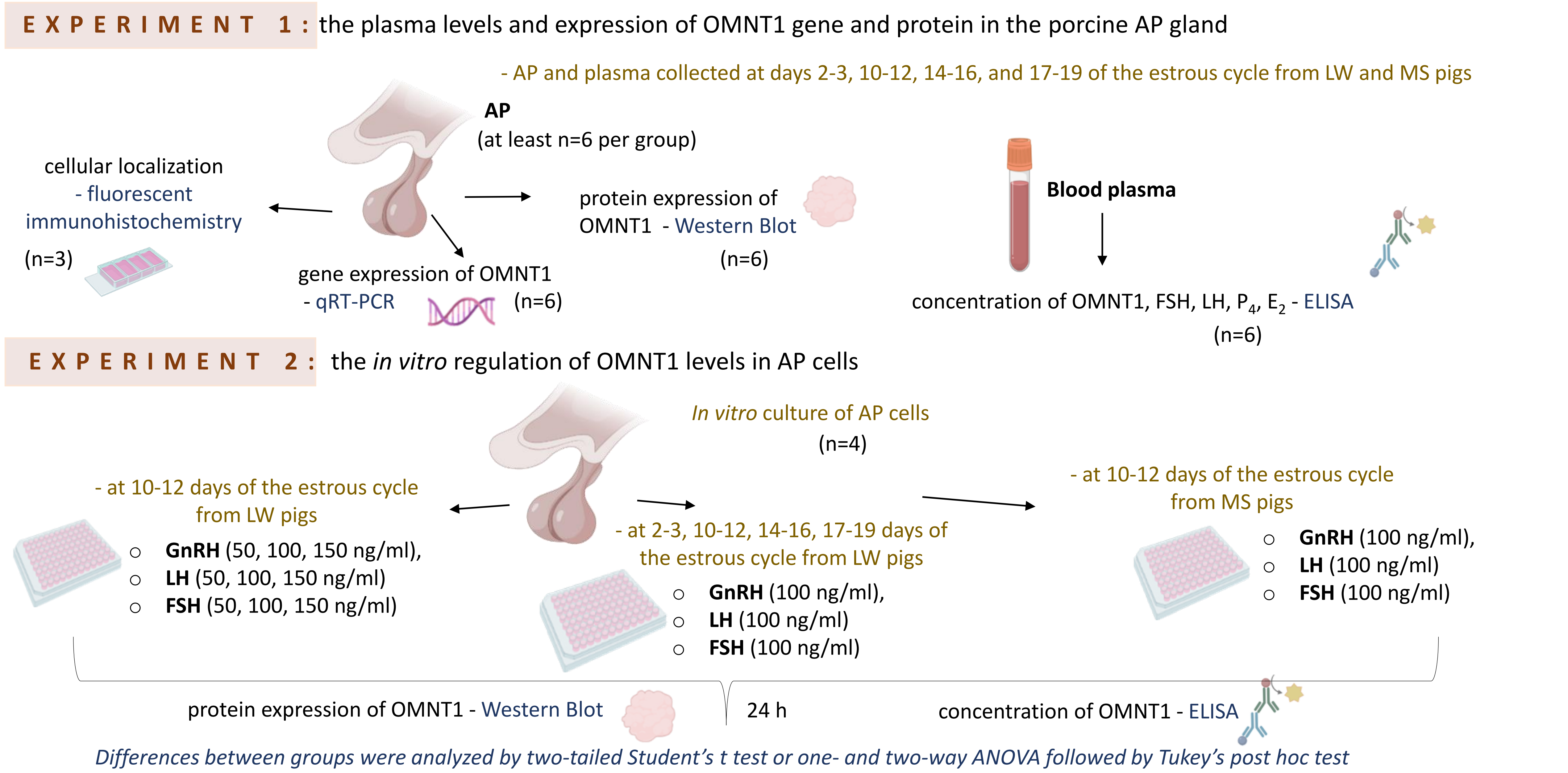

Scheme of the experiments. Blood plasma and the anterior pituitary (AP) gland were during the estrous cycle of Large White (LW) and Meishan (MS) pigs. The gene and protein expression of omentin-1 (OMNT) were analyzed by qRT-PCR and western blot, respectively. Cellular immunolocalization of OMNT1 was studied using immunofluorescence. *In vitro* experiments on AP cells from LW pigs were conducted to show the effect of GnRH, LH, and FSH on OMNT1 expression and concentration in the culture medium. Based on earlier experiments, only one dose and one phase of the estrous cycle were chosen for the *in vitro* culture of AP cells from MS pigs.
